# Meat Consumption and Cognitive Health by *APOE* Genotype

**DOI:** 10.1001/jamanetworkopen.2026.6489

**Published:** 2026-03-19

**Authors:** Jakob Norgren, Adrián Carballo-Casla, Giulia Grande, Anne Börjesson-Hanson, Hong Xu, Maria Eriksdotter, Erika J. Laukka, Sara Garcia-Ptacek

**Affiliations:** 1 Division of Clinical Geriatrics, Department of Neurobiology, Care Sciences and Society, Karolinska Institutet, Stockholm, Sweden; 2 Aging Research Center, Department of Neurobiology, Care Sciences and Society, Karolinska Institutet and Stockholm University, Stockholm, Sweden; 3 Center for Networked Biomedical Research in Epidemiology and Public Health, Madrid, Spain; 4 Stockholm Gerontology Research Center, Stockholm, Sweden; 5 Clinical Trial Unit, Department of Geriatrics, Karolinska University Hospital, Stockholm, Sweden; 6 Theme Inflammation and Aging, Karolinska University Hospital, Stockholm, Sweden

## Abstract

**Importance:**

The apolipoprotein E (*APOE)* ε4 allele increases Alzheimer disease risk. Understanding genotype-specific dietary needs could inform more personalized prevention strategies.

**Objective:**

To test the hypothesis that higher meat consumption may be associated with cognitive health benefits in individuals with *APOE* genotypes ε3/ε4 and ε4/ε4 (*APOE34/44*) and to examine whether this association differs from that in other genotypes.

**Design, Setting, and Participants:**

This population-based cohort study used panel data analyses conducted in January 2025 to January 2026 over 15 years of follow-up in the Swedish National Study on Aging and Care–Kungsholmen (SNAC-K), using strategies aligned with causal inference principles. Recruitment was done in 2001 to 2004 among adults without dementia aged 60 years or older.

**Exposures:**

The primary exposure was total meat consumption in grams per total kilocalories assessed via validated food frequency questionnaires. The secondary exposure was the ratio of processed to total meat.

**Main Outcomes and Measures:**

Global cognitive trajectory, measured as change in *z* score per 10 years, was analyzed by linear regression. Incident dementia was analyzed using Fine and Gray subdistribution hazard ratios (sHRs), treating nondementia death as a competing risk.

**Results:**

Among 2157 older adults without dementia (mean [SD] age 71.2 [9.2] years; 1337 female [62.0%]), 1680 participants had longitudinal cognition data and 569 participants (26.4%) had *APOE34/44* genotypes. During follow-up, 296 participants developed dementia and 690 died without dementia. Among participants with *APOE34/44* genotypes, higher total meat consumption (top vs bottom quintile) was associated with better cognitive trajectories (β = 0.32; 95% CI, 0.07 to 0.56; *P* = .01) and reduced dementia risk (sHR, 0.45; 95% CI, 0.21 to 0.95; *P* = .04). No associations were found in participants with *APOE22/23/24/33* genotypes (cognitive trajectory: β = –0.11; 95% CI, –0.27 to 0.06; *P* = .20; dementia: sHR, 0.95; 95% CI, 0.57 to 1.61; *P* = .86). *P* values for *APOE* interaction were .004 for cognition and .10 for dementia. In the top quintile of meat consumption, dementia risk and cognitive decline were similar between *APOE* strata. A higher ratio of processed to total meat was unfavorably associated with dementia (sHR, 1.14; 95% CI, 1.01 to 1.29; *P* = .04), showing no *APOE* interaction and no substantial difference between unprocessed red meat and poultry. Post hoc analyses suggested concordant *APOE* interaction for all-cause mortality (unprocessed meat exposure, *APOE34/44*: HR, 0.85; 95% CI, 0.73 to 0.99; *P* = 0.04; *P* for interaction = .03).

**Conclusions and Relevance:**

In this study, higher meat consumption was associated with better cognitive trajectories and lower dementia risk among individuals with *APOE34/44* genotypes. The expected cognitive disadvantage among individuals with *APOE34/44* genotypes was not observed at high meat consumption, suggesting clinical and public health relevance.

## Key Points

**Question:**

Is higher meat consumption associated with better cognitive health among individuals with *APOE* genotypes ε3/ε4 and ε4/ε4, and does this association differ from that observed in other genotypes?

**Findings:**

In this cohort study among 2157 older adults without dementia, higher total meat consumption was associated with slower cognitive decline and a reduced dementia risk among older adults with *APOE* ε3/ε4 and ε4/ε4 genotypes. Interactions by *APOE* genotype were observed for trajectories of global cognition and episodic memory.

**Meaning:**

These findings suggest that higher meat consumption than conventionally recommended may be associated with benefits in a genetically defined subgroup comprising approximately one-quarter of the global population.

## Teaser

This cohort study investigates the association of meat consumption with risk of cognitive decline and dementia among older adults with *APOE* genotypes ε3/ε4 and ε4/ε4.

## Introduction

Apolipoprotein E (*APOE*) is the predominant genetic risk modifier for Alzheimer disease, with 3 variants (ε4/ε3/ε2 alleles), yielding 6 different genotypes.^1^ Empirical findings by us^2^ and others^3,4^ indicate that *APOE* modifies response to dietary factors, which may be explained by evolutionary aspects.^5,6^ *APOE* ε4 (*APOE4*) originated 1 million to 6 million years ago and represents the ancestral human form; *APOE3* is thought to have emerged approximately 200 000 years ago, while *APOE2* arose more recently.^5^ The most common genotype, ε3/ε3 (*APOE33*), accounts for more than 50% of *APOE* genotypes in most ethnic populations.^7^ *APOE44* confers the highest Alzheimer risk, with odds ratios compared with *APOE33* varying by ancestry. They approach 30 in East Asian, 13 in White, 6 in Black, and 4 in Hispanic populations, while corresponding odds ratios for *APOE34* are 4, 3, 2, and 2, respectively.^8^

**Figure 1.**
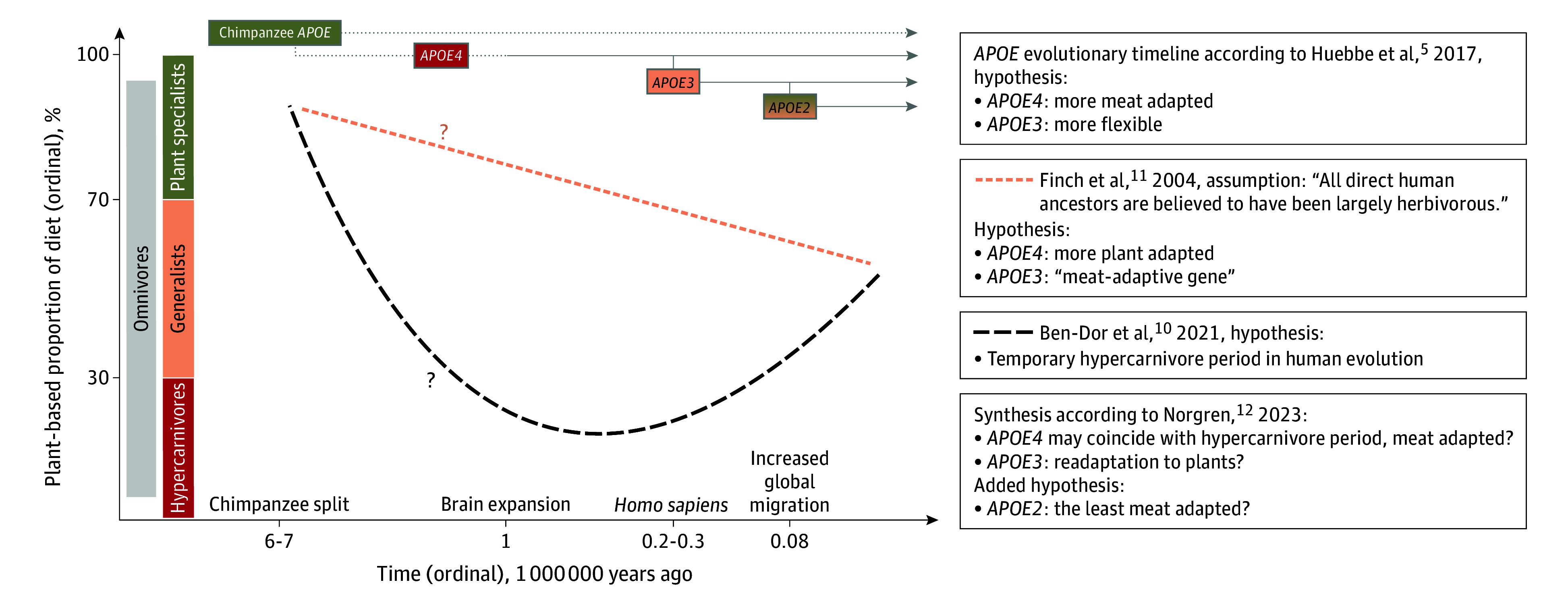
Overview of Hypotheses on Dietary Habits During Human Evolution and *APOE*-Specific Dietary Adaptations In contrast to the Finch et al^11^ suggestion that the *APOE3* genotype may be meat adaptive, Huebbe et al^5^ argue that *APOE4* may provide adaptation to meat consumption, citing resistance to parasitic infections, reduced detoxification capacity of plant compounds, and preferential binding to larger lipoproteins rather than high-density lipoprotein. Plant specialists (consuming a >70% plant-based diet), generalists (30%-70%), and hypercarnivores (<30%) are defined according to Ben-Dor et al,^10^ 2021. Figure adapted from Norgren,^12^ 2023. Curves represent ordinal approximations based on interpretation of referenced sources. The chimpanzee apolipoprotein E (ApoE) protein is monomorphic and believed to function more like human ApoE3 and ApoE2 than ApoE4.^5,13^

The plant-to-animal food ratio is heterogenous in ancestral human diets, but a general shift toward increased meat consumption occurred 2.5 million years ago.^9^ A hypothesis by Ben-Dor et al^10^ states that the shift toward a lower plant-based proportion was not linear but J shaped (see our interpretation in Figure 1).^5,10,11,12,13^ Those authors propose that a hypercarnivorous period occurred a few million years ago,^10^ which may correspond to *APOE4* emergence,^2^ followed by a return toward more plant-based diets in the last hundreds of thousands of years, coinciding with *APOE3* emergence. Conflicting hypotheses suggest that *APOE4* may provide more^5^ or less^11^ adaptation to higher meat consumption. The former view is more compatible with the J-shaped hypothesis and implies that *APOE3* may confer increased metabolic flexibility, potentially enabling evolutionary readaptation to a more plant-based, omnivorous diet.^12^

In our previous work among older adults at risk for dementia, a *higher-carbohydrate-fiber-lower-fat-protein* composite score (proxy for the plant-to-animal ratio) was negatively associated with global cognition among individuals with *APOE34/44* genotypes. A lack of an association for *APOE33* supported the notion of metabolic flexibility, whereas *APOE2* carriers exhibited positive trends, suggesting opposing adaptation relative to *APOE4*. Building on these findings, we outlined our hypothesis on *APOE-*specific dietary adaptation, referencing the Bradford Hill viewpoints on decision-making from observational data.^12,14^ One supporting perspective is the lower prevalence of *APOE4*, suggesting adverse selection, in agricultural regions.^15^ In Europe, *APOE4* allele frequency decreases gradually from up to 27% in the north to as low as 4% in the south.^16^

Reviews of studies on meat consumption in association with cognitive health outcomes indicate that findings and methodologies are inconsistent.^17,18,19^ Some studies reported *APOE* interaction analyses, although outcomes were not statistically significant.^20,21^ Our aim for this study was to estimate the effect of meat consumption on global cognition and dementia incidence among individuals with genotypes ε3/ε4 and ε4/ε4 (*APOE34/44*) compared with individuals with *APOE22/23/24/33* (non-*APOE34/44*) genotypes in a population-based cohort of older adults. As stated prior to data access,^12^ we hypothesized that higher meat consumption would be associated with distinct benefits among individuals with *APOE34/44* genotypes.

## Methods

### Study Population

The Swedish National Study on Aging and Care–Kungsholmen (SNAC-K) is an ongoing, longitudinal, population-based study targeting individuals aged 60 years or older in an urban area of Stockholm.^22^ In the first wave (2001-2004), 3363 of 5111 randomly selected individuals were enrolled; 2157 participants met the inclusion criteria for this analysis: baseline data on diet, cognition, and *APOE* status and no dementia at baseline (see flowchart in eFigure 1 in Supplement 1).

This cohort study was approved by the Swedish Ethical Review Authority. Written informed consent was obtained from all participants or proxies for those cognitively impaired. The study adhered to the Declaration of Helsinki and the Strengthening the Reporting of Observational Studies in Epidemiology—Nutritional Epidemiology (STROBE-Nut) reporting guideline.

### Study Design

We used panel data with repeated exposure and outcome measures over a period of up to 15 years. Participants were assessed every 6 years until age 78 years and every 3 years thereafter. Cognitive performance and dementia were evaluated at each visit; dietary assessments were conducted at baseline and at 3- and 6-year follow-ups.

A triangulation approach was used to examine cognition, integrating longitudinal between-participant outcomes (years 0-15) as the primary analysis, sensitivity analyses on within-participant outcomes (years 0-6), and a cross-sectional baseline analysis to account for individuals lost before follow-up. Primary analyses were conceptualized as a parallel-group target trial,^23^ with mean dietary intake across follow-ups representing long-term exposure; within-participant analyses were framed as crossover trials, as detailed in a previous study^24^ and eMethods 1 in Supplement 1. Time-to-event analyses estimated the association of baseline diet (to preserve temporality) with incident dementia.

### Exposure Variables

Dietary intake was assessed using validated, semiquantitative, 98-item food-frequency questionnaires capturing diet over the previous year.^25^ The primary exposure was total meat consumption (grams per total kilocalories, as explained in eFigure 2 in Supplement 1). Secondary exposures were the processed-to-total meat ratio and the log ratio of unprocessed red meat to poultry (correlation matrix for exposures shown in eTable 1 in Supplement 1). Processed meat was defined as meat transformed through salting, curing, fermentation, smoking, or other processes. Cognitive trajectories were analyzed from mean consumption levels across follow-ups, with sensitivity analyses using baseline and last measurement, respectively. Dementia analyses used baseline values. Data on dietary supplements were not available.

### Outcome Variables

Global cognition was calculated as the mean *z* score across 4 domains: episodic memory (free recall and recognition), semantic memory (vocabulary), verbal fluency (animals and professions), and perceptual speed (digit cancellation and pattern comparison).^26^ Scores were standardized to baseline values of the study sample before creation of a composite score of global cognition for all individuals with data in at least 2 cognitive domains (2157 participants). For primary analyses, a longitudinal cognitive trajectory (change in *z* score per year) was precalculated for each participant and domain separately using linear regression, with linear time as the factor estimating log-transformed cognitive outcomes. Baseline data from all domains and at least 1 follow-up in each domain were specified as inclusion criteria (1680 participants).

Dementia was diagnosed per *Diagnostic and Statistical Manual of Mental Disorders* (Fourth Edition) (*DSM-IV*) criteria, based on medical and drug history, general and neurological exams, and cognitive tests (eg, Mini-Mental State Examination [MMSE] and clock drawing). Daily living independence was also assessed. Diagnoses followed a 3-step process: initial physician assessment, independent review of available information by a second physician, and adjudication by senior neurologists (including G.G.) if discrepant. For participants who died between follow-ups, clinical records and death certificates were reviewed.^27^

### Covariates

Covariate selection followed recommendations for causal inference and used baseline values for adjustment assuming negligible exposure feedback mechanisms.^23,28^ For diet covariates, the mean across visits was used, when applicable, to align with the factor of interest. The model included age, sex, education, *APOE* status, living arrangements, lifelong occupation type, physical activity level, current smoking status, alcohol intake, total energy intake, and Alternative Healthy Eating Index (AHEI) score,^29^ excluding meat items, as a marker for adherence to dietary guidelines. Morbidity^30^ (<2, 2-5, or >5 chronic diseases) and baseline cognition (except on dementia analyses, where this was considered to be a mediating pathway) were also included. The composite morbidity variable was used for simplicity given that separate adjustment for multiple diagnoses (hypertension, dyslipidemia, diabetes, cardiovascular or cerebrovascular disease, obesity, anemia, and depression) yielded similar results. Because no covariate beyond age substantially modified estimates, reporting is restricted to our primary model. A sensitivity analysis explored 6 macronutrient parameters as potential mediators. For missing covariates (<1% for all), the mean or mode (for categorical variables) was imputed.

### Effect Modifiers

*APOE* was determined by standard methods^26^ and primarily dichotomized as *APOE34/44* vs non-*APOE34/44* genotypes, consistent with our prior work.^2^ Ancillary stratifications explored a hypothesized interaction gradient (*APOE22-23-24-33-34-44*).^12^

### Statistical Analysis

Between-participant analyses on cognition used linear regression, with quintile-based analyses in primary analyses to assess potential nonlinearity. A fixed-effects model was used for within-participant analyses.^31^ Robust standard errors were estimated. Dementia analyses used Fine and Gray models, treating nondementia death as a competing risk. Proportional subdistribution hazard assumptions were confirmed graphically using cumulative incidence curves. Dementia onset was defined as the midpoint between the last known dementia-free day and the day of diagnosis. Time zero was set at baseline, and exit was defined as the earliest occurrence of dementia onset, death, or, for survivors without dementia, the day after the last follow-up. Exit was truncated to 15.5 years from baseline for 37 participants, who were without dementia, who had their last follow-up beyond that time to mitigate potential selection bias. Given that attrition was not associated with *APOE* or meat consumption (Table; eFigure 3 in Supplement 1), meaningful bias from attrition was considered unlikely.

**Table.** Baseline Participant Characteristics by Meat Consumption Quintile and *APOE* Genotype

Characteristic^a^	Participants, No. (%)^b^					
	Q1	Q2	Q3	Q4	Q5	Full sample
Participants, No.						
* APOE34/44*	108	131	102	100	128	2157
Other genotype	324	300	330	331	303	
Age, mean (SD), y						
* APOE34/44*	71.8 (9.3)	72.1 (8.7)	71.7 (8.9)	68.9 (8.7)	67.3 (7.6)	71.2 (9.2)
Other genotype	74.4 (9.7)	73.6 (9.7)	71.4 (8.9)	69.8 (8.9)	68.4 (8.3)	
Sex (females/males)						
* APOE34/44*	77/31 (71.3/28.7)	84/47 (64.1/35.9)	58/44 (56.9/43.1)	55/45 (55.0/45.0)	80/48 (62.5/37.5)	1337/820 (62.0/38.0)
Other genotype	224/100 (69.1/30.9)	185/115 (61.7/38.3)	201/129 (60.9/39.1)	204/127 (61.6/38.4)	169/134 (55.8/44.2)	
Time to last follow-up visit, mean (SD), y						
Overall						
* APOE34/44*	8.6 (4.9)	8.6 (4.9)	8.0 (5.2)	8.5 (4.7)	9.3 (5.1)	8.8 (5.1)
Other genotype	8.5 (5.4)	8.9 (5.2)	9.0 (5.3)	9.4 (4.9)	8.7 (5.0)	
In participants included in cognition analyses						
No. with data	323	329	344	346	338	1680
* APOE34/44*	10.4 (3.6)	10.5 (3.6)	10.4 (3.5)	10.1 (3.3)	11.3 (3.3)	10.8 (3.6)
Other genotype	10.8 (3.9)	10.8 (3.8)	11.0 (3.7)	11.0 (3.4)	10.5 (3.5)	
Global cognition, mean (SD), *z* score						
* APOE34/44*	–0.06 (1.03)	–0.07 (0.87)	–0.08 (0.93)	0.10 (1.06)	0.20 (0.98)	0 (1)
Other genotype	–0.12 (1.08)	–0.17 (1.01)	0.10 (0.94)	0.01 (0.96)	0.13 (1.00)	
Education, mean (SD), y						
* APOE34/44*	12.7 (4.3)	12.2 (3.9)	12.7 (5.1)	12.5 (4.1)	12.9 (4.3)	12.4 (4.2)
Other genotype	12.6 (4.4)	11.7 (3.9)	12.3 (4.2)	12.2 (3.9)	12.6 (4.0)	
Living arrangements (not alone)						
No. with data	432	430	431	429	428	2150
* APOE34/44*	186 (40.7)	151 (46.6)	163 (53.9)	156 (65.7)	128 (46.8)	1066 (49.6)
Other genotype	138 (42.6)	148 (49.5)	166 (50.5)	174 (52.7)	174 (57.6)	
Occupation (manual)						
No. with data	432	430	431	429	428	2150
* APOE34/44*	19 (17.6)	30 (22.9)	15 (14.7)	15 (15.2)	23 (18.3)	396 (18.4)
Other genotype	69 (21.3)	60 (20.1)	63 (19.1)	53 (16.1)	49 (16.2)	
Physical activity^c^						
Low						
* APOE34/44*	18 (16.7)	25 (19.1)	19 (18.6)	21 (21.0)	28 (21.9)	458 (21.2)
Other genotype	70 (21.6)	70 (23.3)	62 (18.8)	69 (20.8)	76 (25.1)	
Mid						
* APOE34/44*	66 (61.1)	69 (52.7)	51 (50.0)	58 (58.0)	67 (52.3)	1141 (52.9)
Other genotype	176 (54.3)	148 (49.3)	184 (55.8)	173 (52.3)	149 (49.2)	
High						
* APOE34/44*	24 (22.2)	37 (28.2)	32 (31.4)	21 (21.0)	33 (25.8)	558 (25.9)
Other genotype	78 (24.1)	82 (27.3)	84 (25.5)	89 (26.9)	78 (25.7)	
Tobacco smoker (current)						
No. with data	429	429	430	430	426	2144
* APOE34/44*	12 (11.1)	16 (12.4)	10 (9.9)	14 (14.1)	20 (15.9)	279 (13.0)
Other genotype	36 (11.2)	30 (10.0)	35 (10.6)	47 (14.2)	59 (19.7)	
BMI, mean (SD)						
* APOE34/44*	24.9 (3.6)	25.7 (3.5)	25.7 (3.4)	26.5 (4.3)	26.6 (4.0)	26.0 (4.0)
Other genotype	25.2 (3.9)	25.9 (4.2)	25.7 (3.6)	26.2 (4.3)	26.9 (3.9)	
Systolic blood pressure, mean (SD), mm Hg						
No. with data	430	430	432	430	431	2153
* APOE34/44*	143 (21)	146 (19)	143 (22)	141 (17)	144 (16)	144 (19)
Other genotype	145 (19)	144 (19)	144 (21)	145 (19)	144 (19)	
HbA_1c_ level, mean (SD), %						
No. with data	427	430	425	423	421	2126
* APOE34/44*	4.53 (0.43)	4.53 (0.43)	4.67 (0.86)	4.66 (0.80)	4.46 (0.47)	4.57 (0.68)
Other genotype	4.54 (0.57)	4.56 (0.71)	4.55 (0.75)	4.61 (0.73)	4.65 (0.75)	
Total cholesterol, mean (SD), mg/dL						
No. with data	426	429	426	423	420	2124
* APOE34/44*	246 (43)	243 (40)	233 (40)	241 (40)	236 (41)	234 (43)
Other genotype	230 (43)	233 (44)	231 (43)	231 (42)	233 (44)	
Chronic diseases, mean (SD), No.						
* APOE34/44*	3.8 (2.1)	3.6 (2.0)	3.4 (2.1)	3.4 (2.1)	3.4 (2.1)	3.6 (2.2)
Other genotype	3.7 (2.2)	3.8 (2.3)	3.5 (2.2)	3.5 (2.4)	3.5 (2.3)	
Diabetes						
* APOE34/44*	5 (4.6)	5 (3.8)	10 (9.8)	10 (10.0)	7 (5.5)	166 (7.7)
Other genotype	15 (4.6)	21 (7.0)	24 (7.3)	32 (9.7)	37 (12.2)	
Total energy intake, mean (SD), kcal/d						
* APOE34/44*	2028 (660)	1972 (595)	2037 (659)	1993 (622)	1783 (576)	1970 (651)
Other genotype	2022 (685)	1984 (611)	2029 (701)	1973 (637)	1864 (653)	
Carbohydrates, mean (SD), E%						
* APOE34/44*	46.6 (6.4)	45.6 (6.2)	43.6 (6.3)	43.3 (6.5)	42.1 (5.8)	44.2 (6.8)
Other genotype	47.3 (7.7)	44.8 (6.8)	44.3 (6.3)	43.7 (6.0)	41.0 (6.3)	
Fat, mean (SD), E%						
* APOE34/44*	33.3 (6.7)	34.3 (7.0)	35.2 (6.9)	34.8 (7.1)	34.8 (6.2)	34.6 (6.9)
Other genotype	33.2 (8.0)	35.2 (7.1)	34.4 (6.6)	34.6 (6.3)	35.8 (6.6)	
Protein, mean (SD), E%						
* APOE34/44*	13.3 (2.5)	13.5 (2.1)	13.9 (2.1)	14.4 (2.3)	16.0 (2.4)	14.2 (2.4)
Other genotype	13.0 (2.3)	13.3 (2.1)	14.0 (2.1)	14.6 (2.1)	15.9 (2.3)	
Fiber, mean (SD), g						
* APOE34/44*	27.2 (10.4)	25.9 (10.1)	25.2 (9.6)	23.8 (8.9)	22.5 (9.2)	25.2 (10.3)
Other genotype	28.0 (12.5)	24.7 (9.8)	26.2 (10.6)	24.8 (9.1)	22.4 (9.5)	
Alcohol, mean (SD), E%						
* APOE34/44*	4.2 (4.3)	4.1 (4.0)	5.0 (5.1)	5.2 (4.7)	4.7 (4.4)	4.5 (4.4)
Other genotype	3.8 (5.0)	4.3 (4.4)	4.7 (3.9)	4.7 (4.0)	5.0 (4.4)	
SFA-to-PUFA ratio, mean (SD), log, *z*						
* APOE34/44*	0.00 (1.24)	0.03 (1.00)	–0.02 (0.90)	0.00 (0.87)	–0.22 (0.83)	0 (1)
Other genotype	0.24 (1.25)	0.05 (1.02)	–0.06 (0.94)	–0.05 (0.89)	–0.10 (0.83)	
Meat consumption, mean (SD), g/14 000 kcal^d^						
Total						
* APOE34/44*	215 (97)	398 (29)	509 (32)	642 (48)	934 (214)	539 (264)
Other genotype	221 (98)	395 (33)	511 (33)	642 (46)	928 (220)	
Red meat, unprocessed						
* APOE34/44*	114 (70)	199 (63)	255 (71)	310 (87)	490 (216)	273 (165)
Other genotype	109 (71)	203 (63)	260 (73)	326 (96)	468 (189)	
Poultry, unprocessed						
* APOE34/44*	52 (47)	74 (50)	81 (51)	115 (77)	163 (124)	98 (96)
Other genotype	54 (47)	72 (46)	92 (58)	109 (82)	168 (170)	
Processed meat						
* APOE34/44*	50 (51)	124 (61)	174 (81)	217 (98)	280 (170)	168 (134)
Other genotype	57 (56)	121 (71)	159 (84)	207 (102)	293 (192)	
Processed-to-total meat ratio, mean (SD), %						
No. with data	429	431	432	431	431	2154
* APOE34/44*	21.0 (20.2)	31.5 (15.6)	34.0 (15.6)	33.9 (15.1)	30.0 (15.9)	30 (18)
Other genotype	23.9 (22.5)	30.5 (17.5)	31.1 (16.1)	32.2 (15.6)	31.6 (18.2)	
AHEI score (maximum = 110), mean (SD)						
* APOE34/44*	66 (10)	63 (10)	61 (9)	59 (11)	61 (10)	62 (10)
Other genotype	64 (10)	62 (9)	63 (9)	60 (9)	58 (9)	

Abbreviations: AHEI, Alternative Healthy Eating Index; BMI (body mass index; calculated as weight in kilograms divided by height in meters squared); SFA/PUFA, saturated/polyunsaturated fatty acids (log ratio).

SI conversion factors: To convert cholesterol to millimoles per liter, multiply by 0.0259; HbA_1c_ to proportion of total hemoglobin, multiply by 0.01;

^a^ There were missing data for 16 participants for BMI, 4 participants for systolic blood pressure, 31 participants for HbA_1c_, 33 participants for total cholesterol, 7 participants for living arrangements, 7 participants for occupation, 13 participants for tobacco smoking, and 3 participants for the ratio of processed to total meat (due to 0 total meat consumption).

^b^ Sample size per quintile is indicated for *APOE34/44* and other genotypes (*APOE22/23/24/33*).

^c^ Physical activity was categorized as low (light or moderate-to-intense exercise ≤2 to 3 times/month), high (moderate-to-intense exercise several times/week), or mid (all other levels).

^d^ Meat consumption is expressed as grams per 14 000 kcal (equivalent to weekly consumption for a 2000 kcal/d diet).

To address competing risk and health in broader terms, some post hoc analyses were conducted. All-cause mortality was examined by Cox regression (eMethods 2 in Supplement 1), and associations between meat consumption and some biomarkers were studied by linear regression. Replacement analyses for individual food groups applied log-ratio transformations.^32^

A 2-sided significance level of 5% was used for primary analyses, which were prespecified; therefore, no adjustment for multiple comparisons was performed. Ancillary analyses were exploratory and not intended for formal hypothesis testing; such reporting is primarily graphical. Analyses were performed in January 2025 to January 2026 using Stata statistical software version 18 (StataCorp).

## Results

### Description of Participants

Among 2157 participants (mean [SD] age 71.2 [9.2] years; 1337 female [62.0%]), the prevalence of *APOE34/44* was 569 participants (26.4%). Baseline characteristics across quintiles of meat consumption by *APOE34/44* genotype are presented in the Table and eFigures 4 to 5 in Supplement 1. Status at censoring, showing 296 patients with dementia and 924 deaths, is described in eTable 2 in Supplement 1. A total of 690 patients died without dementia. *APOE* distribution and other characteristics are detailed in eTable 3 in Supplement 1, including a separate column for the subsample of 1680 participants with cognitive trajectories, showing minimal differences from the full sample.

At baseline, 88 participants scored less than 27 on the MMSE and were excluded in sensitivity analyses, alongside participants with dementia within 3 years. Of participants with an MMSE score less than 27, a total of 87 scored in the range of 23 to 26, while 1 participant scored 20 and 22 participants had an *APOE34/44* genotype. A total of 13 food records were collected after dementia diagnosis at follow-up. Excluding these records had a negligible effect on cognitive results, and given that dementia analyses used baseline diet, they were not affected.

### Cognition Analyses

**Figure 2.**
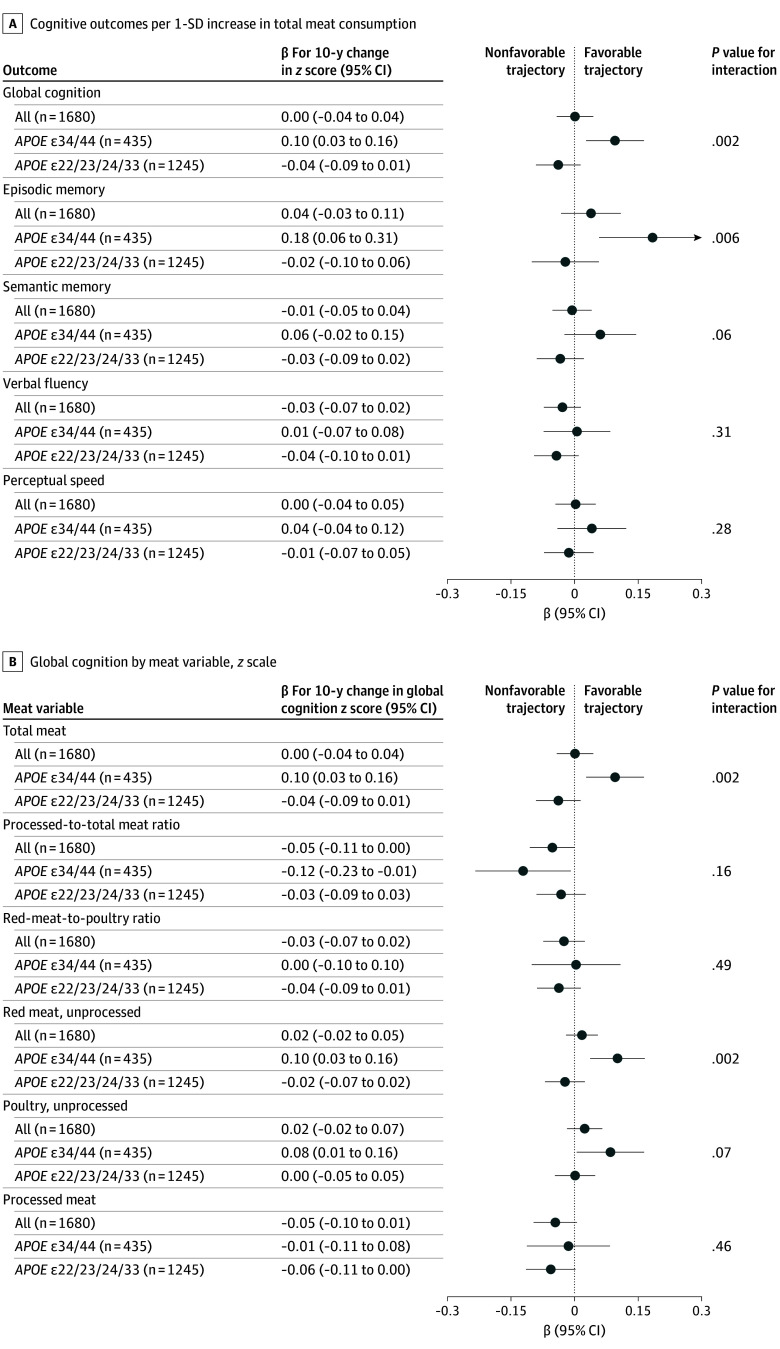
Forest Plots of Linear Associations of Meat Consumption With Cognitive Trajectories A, Estimates per composite cognitive outcome and cognitive subdomain as the outcome. B, Estimates by different meat variable as the exposure. Both analyses use linear regression between meat exposure (in grams per kilocalorie, *z* transformed) and cognitive trajectories (change in *z* score/y multiplied by 10) adjusted for age, sex, education, *APOE* status, living arrangements, occupation type, physical activity, smoking, alcohol intake, total energy intake, Alternative Healthy Eating Index score (calculated without meat items), number of chronic diseases, and baseline cognition. One SD equals the following consumption levels (standardized for 2000 kcal/d intake): 264 g/week for total meat consumption, 165 g/week for unprocessed red meat, 96 g/week for poultry, and 134 g/week for processed meat. *P* values are given for the interaction between meat variables and *APOE* status.

**Figure 3.**
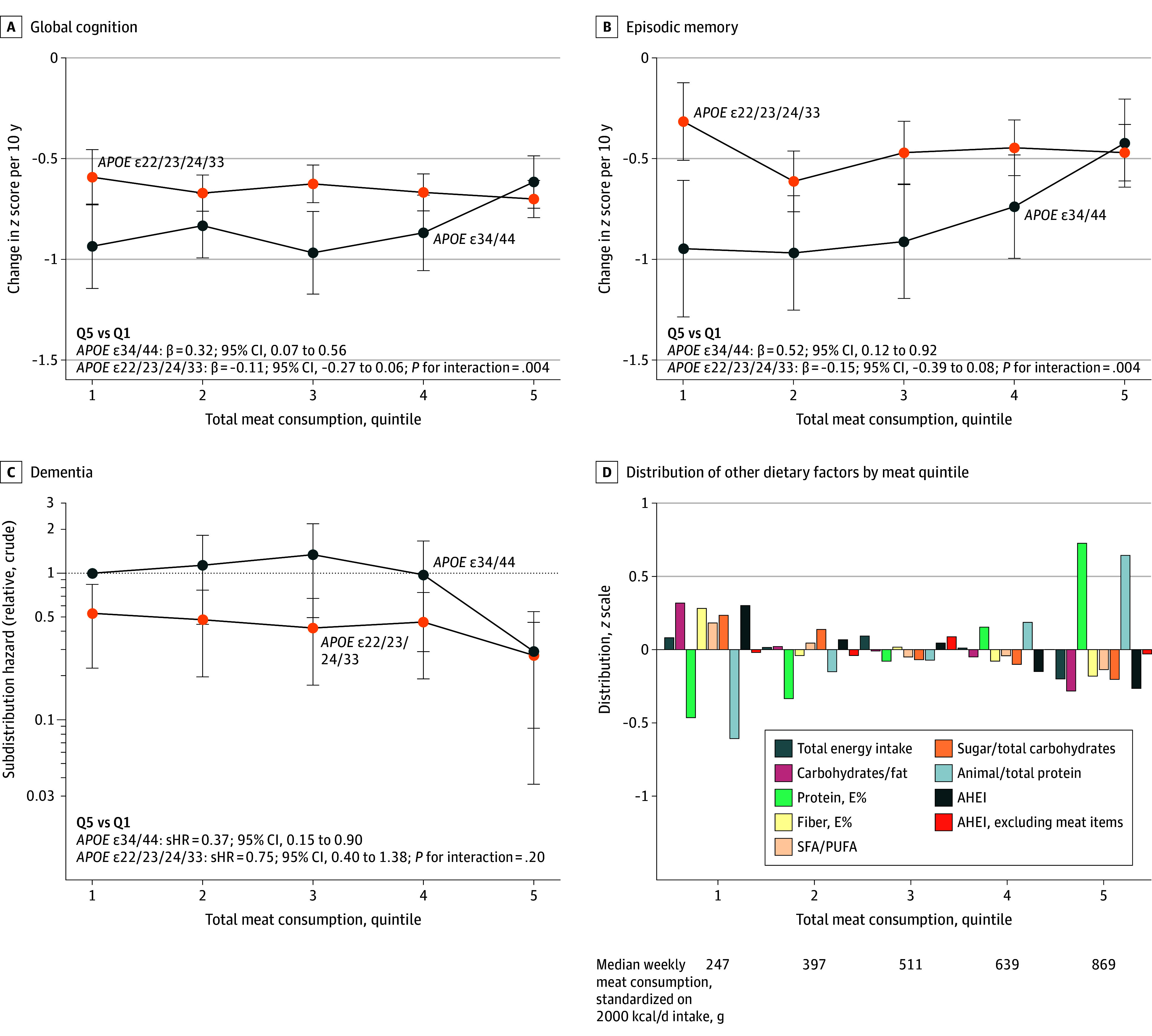
Line Graphs of Quintile-Based Associations of Meat Consumption With Cognitive Health Outcomes The figure displays 3 cognitive outcomes: global cognition (A), episodic memory (B), and dementia incidence (C), analyzed in the same subsample (1680 participants with ≥1 cognitive follow-up) by quintile (Q) of total meat consumption. Q assignment is based on weight per total energy intake. Linear regression (A and B) was adjusted for age, sex, education, *APOE* status, living arrangements, occupation type, physical activity level, smoking status, alcohol intake, total energy intake, Alternative Healthy Eating Index (AHEI; calculated without meat items when used as a covariate) score, baseline cognition, and number of chronic diseases. Similar adjustments, except for baseline cognition, were applied to subdistribution hazard ratios (sHRs) in panel C, whereas plotted values are crude to enhance scaling. D, Distributions of other dietary factors by meat quintile are illustrated among all 2157 participants. No factors changed results substantially when added as covariates (eFigure 8 in Supplement 1). As a reference, the shown consumption levels in Q3 to Q5 clearly exceed the Nordic Nutrition Recommendations,^33^ 2023. *P* values are given for the interaction between exposure (Q5 vs Q1) and *APOE* genotype. E% indicates energy percentage; SFA/PUFA, saturated/polyunsaturated fatty acids.

Linear analyses for total meat consumption and cognitive trajectories are shown in Figure 2A, with meat types analyzed in Figure 2B. Higher meat consumption was associated with favorable trajectories of global cognition and episodic memory for *APOE34/44* but not for non-*APOE34/44* genotypes, with interactions. Global cognitive change per 10 years by total meat quintile is illustrated in Figure 3A.^33^ For *APOE34/44* in the top quintile (Q5), the trajectory was better than Q1 (β = 0.32; 95% CI, 0.07 to 0.56; *P* = .01) and similar to non-*APOE34/44* genotypes regardless of quintile. In exploratory analyses, Q5 was also better compared with Q2 (*P* = .04), Q3 (*P* = .004), and Q4 (*P* = .03). The largest magnitude was observed for episodic memory (Figure 3B). No association was found with cognitive trajectory in participants with non-*APOE34/44* genotypes (β = –0.11; 95% CI, –0.27 to 0.06; *P* = .20) for Q5 vs Q1. *APOE* interaction (*P* for interaction for cognitive decline = .004) was robust across sensitivity analyses and more pronounced among females, participants aged 72 years and younger, and individuals with higher hemoglobin A_1c_ (HbA_1c_) levels, lower AHEI scores, and Cardiovascular Risk Factors, Aging, and Dementia (CAIDE) risk scores^34^ of 6 or greater (eFigure 6 in Supplement 1). Complete *APOE* stratification supported our primary dichotomization (eFigure 7 in Supplement 1). Mediation analyses on macronutrient parameters showed minimal changes (eFigure 8 in Supplement 1). Triangulation approaches (within-participant and cross-sectional baseline analyses) yielded results in the same direction as the primary analysis (eFigure 9 in Supplement 1). Comparing individuals who did vs did not develop dementia, *APOE* interactions remained similar, and dietary changes did not differ, collectively suggesting that reverse causation was unlikely (eFigure 10 in Supplement 1).

A higher ratio of processed to total meat was associated with a worse cognitive trajectory in *APOE34/44* only (β = –0.12; 95% CI, –0.23 to –0.01; *P* = .04), although without *APOE* interaction. The ratio of red meat to poultry was not associated with cognitive trajectory (Figure 2B).

### Dementia Analyses

**Figure 4.**
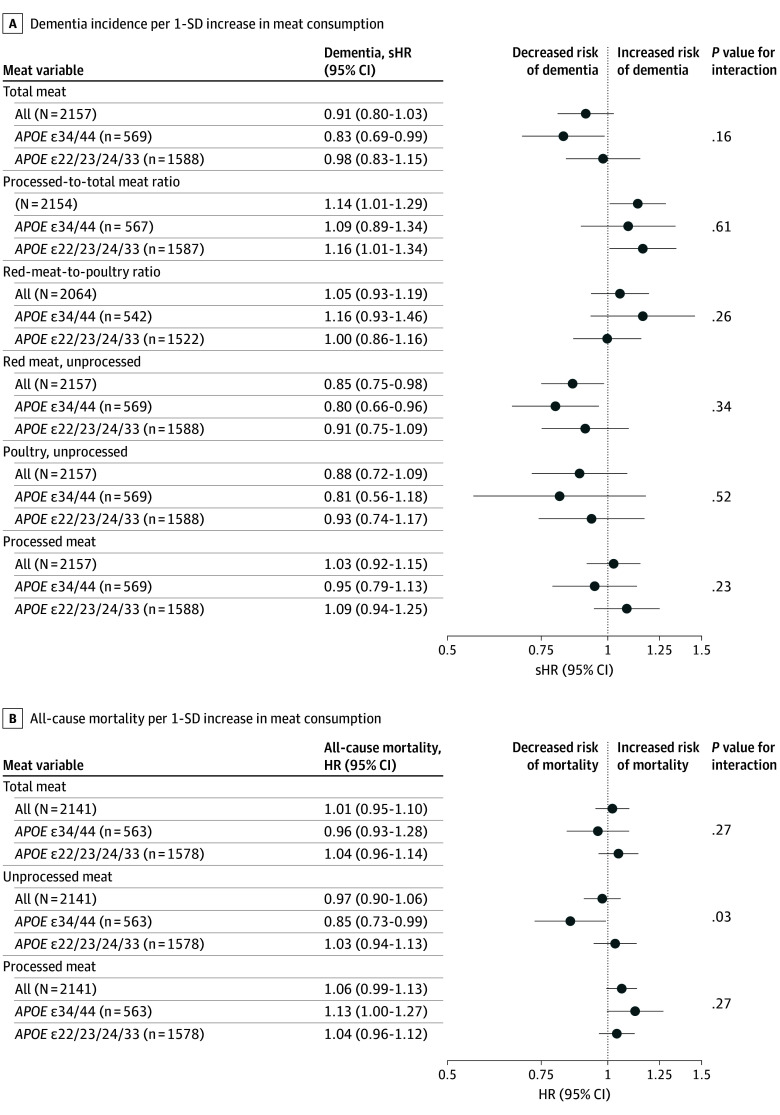
Forest Plots of Dementia and All-Cause Mortality Analyses A, Dementia incidence was analyzed using Fine and Gray models, with nondementia death as a competing risk. One SD equals the following consumption levels (standardized for 2000 kcal/d intake): 264 g/week for total meat, 165 g/week for unprocessed red meat, 96 g/week for poultry, 134 g/week for processed meat. B, A post hoc analysis on all-cause mortality was conducted to guide interpretations of cognitive outcomes using Cox proportional hazards regression, excluding deaths occurring within the first year after baseline. Unprocessed red meat and poultry were grouped as *unprocessed meat* after concluding that the ratio between those meat types was not associated with the outcome (hazard ratio [HR], 1.01; 95% CI, 0.94-1.08; *P* = .87; *P* for *APOE* interaction = .89). Both analyses accounted for age, sex, education, *APOE* status, living arrangements, occupation type, physical activity level, smoking status, alcohol intake, total energy intake, Alternative Healthy Eating Index score (calculated without meat items), and number of chronic diseases. *P* values are given for the interaction between meat variables and *APOE* genotype. sHR indicates subdistribution hazard ratio.

Subdistribution hazard ratios (sHRs) for dementia incidence aligned with findings on cognitive trajectories. For Q5 vs Q1 of total meat consumption among 2157 participant, sHRs were as follows: 0.72 (95% CI, 0.46-1.11; *P* = .14) in the full sample, 0.45 (95% CI, 0.21-0.95; *P* = .04) for *APOE34/44* genotypes, and 0.95 (95% CI, 0.57-1.61; *P* = .86) for non-*APOE34/44* genotypes (*P* for interaction = .10). For *APOE34/44* compared with non-*APOE34/44* genotypes, the sHR was 2.49 (95% CI, 1.47-4.22; *P* = .001) in Q1, decreasing to 1.17 (95% CI, 0.55-2.45; *P* = .69) in Q5 of total meat. Ancillary quintile analyses are reported in eTable 4 in Supplement 1, with a subsample shown in Figure 3C and cumulative incidence curves in eFigure 11 in Supplement 1. Linear estimates are shown in Figure 4A; these were more robust after excluding individuals with possible cognitive impairment (eFigure 12 in Supplement 1). Quintile-based analyses indicated that for *APOE34/44*, Q5 differed from Q1 through Q4, whereas a slight elevation observed in Q3 did not represent a statistically significant difference vs Q1, Q2, or Q4 (eTable 4 in Supplement 1).

A higher ratio of processed to total meat was associated with increased dementia risk (sHR, 1.14; 95% CI, 1.01-1.29; *P* = .04). This was not modified by *APOE* status (Figure 4A).

### Post hoc Analyses

Findings appeared more robust among females. However, no interaction between meat consumption and sex, regardless of *APOE* genotype, was observed for global cognition (eFigure 6 in Supplement 1) or dementia (eTable 4 in Supplement 1).

Cox regression analyses on all-cause mortality revealed *APOE* interactions consistent with cognitive findings. Specifically, higher unprocessed meat consumption at baseline was associated with reduced mortality among participants with *APOE34/44* genotypes (HR, 0.85; 95% CI, 0.73-0.99; *P* = .04; *P* for interaction = .03), with a trend in the opposite direction for non-*APOE34/44* genotypes (Figure 4B; eFigure 13 in Supplement 1). Similar trends of *APOE* interaction were found for outcomes of cholesterol and HbA_1c_ levels, but not for body mass index or blood pressure levels (eFigure 13 in Supplement 1).

We used the slope between dietary and circulating levels of vitamin B12 as a possible proxy for nutrient absorption to explore a mechanistic hypothesis. A 3-way interaction for *dietary vitamin B12* × *meat consumption* × *APOE status* regressed on vitamin B12 in blood suggested that for participants with *APOE33* genotypes, absorption did not vary across levels of meat consumption. However, for participants with *APOE34/44* genotypes, absorption was greater with higher meat consumption, while the opposite pattern was observed for *APOE2* carriers (eFigure 14 in Supplement 1).

Replacement analyses for individual food groups indicated more favorable cognitive associations in *APOE34/44* genotypes when meat replaced cereals, dairy, oils, and legumes, with no associations for the replacement of eggs, fish, or tubers (eFigure 15 in Supplement 1). Total meat consumption was inversely associated with cereals, dairy, and fruits primarily (eFigure 16 in Supplement 1).

## Discussion

In this population-based cohort study of older adults, higher total meat consumption was associated with favorable cognitive health outcomes for *APOE34/44* genotypes. Findings were consistent for longitudinal analyses of global cognition, episodic memory, and dementia risk, with interactions by *APOE* status for global cognition and episodic memory. None of those estimates were statistically significant in non-*APOE34/44* genotypes. A lower processed-to-total meat ratio was favorably associated with dementia, with no substantial difference between unprocessed red meat and poultry. When meat types were analyzed as fractions of the total diet, associations were observed for unprocessed red meat but not for processed meat; higher consumption of unprocessed red meat was associated with lower dementia risk regardless of *APOE* status. After exclusion of individuals with possible cognitive impairment, this association became more robust and extended to total meat. In post hoc analyses, mortality rates were lower with higher unprocessed meat consumption exclusively among participants with *APOE34/44* genotypes, compatible with findings from a Chinese cohort study.^35^ While exposures that are associated with both dementia and death can yield paradoxical associations (eg, smoking may appear protective against dementia by reducing survival time),^36^ this explanation seems unlikely for *APOE34/44* given the concurrent association with longer survival.

To our knowledge, this is the first study to demonstrate interactions between meat consumption and *APOE* status in the association with cognitive outcomes, in support of a prespecified hypothesis that higher meat consumption may be advantageous in *APOE34/44* genotypes.^12^ However, our findings align with underappreciated patterns in 2 large cohorts. In the UK Biobank (493 888 participants), unprocessed red meat was inversely associated with dementia (*P* = .01), driven by *APOE4* carriers (HR, 0.64 per 50-g/d increase; *P* < .001), with no associations in noncarriers (HR, 0.93; *P* = .59).^21^ In the Nurses’ Health Study (NHS) and Health Professionals Follow-up Study (133 771 participants), supplementary analyses revealed an *APOE4* interaction (*P* < .001) for unprocessed red meat, showing favorable trends among carriers and adverse trends among noncarriers.^37^ These patterns were not emphasized in the original publications, possibly because *APOE* interaction was not the primary focus or because conventional significance thresholds were strictly applied, but they are consistent with our findings. For processed meat, which accounts for approximately one-third of total meat intake across the cohorts discussed and in our study (although measures are not directly comparable), prior studies have reported adverse associations with cognitive health outcomes,^21,37^ which we did not observe. However, a relative advantage of unprocessed over processed meat was shown for dementia.

Intriguingly, among *APOE4* carriers in the NHS, women aged 70 years and older consuming 1 or more servings/d of unprocessed red meat compared with less than 0.50 servings/d had a cognitive advantage of the same magnitude as the disadvantage typically observed in *APOE4* carriers vs noncarriers (approximately 3 years of cognitive aging).^37^ This parallels effect sizes observed in our cohort, where the expected excess risk among participants with *APOE34/44* genotypes was absent in the highest quintile of meat consumption across global cognition, episodic memory (a hallmark feature of Alzheimer pathology^38^), and dementia outcomes. Given that these genotypes account for approximately 70% of Alzheimer dementia cases in Northern Europe and North America,^39^ the absolute number of potentially preventable cases is substantial.

Indirect evidence of a similar *APOE* interaction was implied by reports that the low-meat EAT-Lancet^40^ and Planetary Healthy diets^41^ were favorably associated with cognitive health outcomes only among non–*APOE4* carriers. Notably, the 2025 EAT-Lancet 2.0^42^ cites the NHS and UK Biobank studies discussed previously to support the statement that “red meat has been positively associated with…unhealthy ageing,” a conclusion that is contradicted by our interpretation.

Macronutrient parameters did not mediate our findings, prompting exploration of alternative explanations. Post hoc analyses using a proxy for vitamin B12 absorption suggested distinct *APOE* responses, with better nutrient uptake from meat than from other sources among participants with *APOE34/44* genotypes. These findings raise the possibility that *APOE*-modified health associations may be influenced by the food matrix^43^ or by antinutritive factors in foods replacing meat (primarily cereals and dairy). Indeed, more favorable associations in *APOE34/44* were observed when meat replaced relatively recent additions to the human diet, supporting an evolutionary perspective for future mechanistic research.

Contrary to the Finch et al,^11^ 2004, hypothesis suggesting *APOE3* as the “meat adaptive” allele, our findings suggest that this characteristic fits *APOE4*. However, their hypothesis was based on the assumption that “all direct human ancestors are believed to have been largely herbivorous.”^11^ This conflicts with the hypothesis by Ben-Dor et al,^10^ which implies that a temporary hypercarnivorous period may have coincided with the emergence of *APOE4* in human evolution^12^ (Figure 1). While Finch et al^11^ associated high meat consumption with elevated cholesterol levels and chronic disease risk, our data showed that for participants with *APOE34/44* genotypes, higher meat consumption aligned with lower blood cholesterol levels and a lower ratio of dietary saturated to polyunsaturated fat, a key dietary determinant of cholesterol levels.^44^ This is plausible given that unprocessed meat may contain more unsaturated than saturated fat.^45^

### Strengths and Limitations

Our study’s strengths include a triangulation approach to cognition, showing consistency across between- and within-participants analyses, along with progression to dementia. We adjusted extensively for potential confounders and explored possible reversed causality in sensitivity analyses; however, residual bias cannot be ruled out. Furthermore, we validated our primary *APOE* dichotomization and provided supporting evidence for separating ε24 from ε34/44, in line with previous work,^2^ although results were similar to those of a conventional stratification by *APOE4* carriers. Our study also includes several limitations. One limitation could be potential survival bias; that is, our selection may include particularly resilient individuals. Ethnic ancestry, although not explicitly measured, was homogeneous and predominantly Northern European, and this may limit generalizability. Self-reported dietary data may include errors, but we find that unlikely to have induced bias for *APOE* interactions.

## Conclusions

In this cohort study, we found that the *APOE34/44* group exhibited the anticipated excess risk of cognitive decline and dementia progression compared with participants with other genotypes when consuming meat at levels consistent with current dietary guideline targets. However, this disadvantageous association was absent at higher consumption levels, equivalent to more than twice the target.^33^ Viewed alongside reinterpreted evidence from NHS^37^ and UK Biobank^21^ focusing on unprocessed meat, these findings point to a consistent gene-diet interaction, with important implications for public health. Results reinforce the urgency of investing in precision nutrition research with a focus on *APOE*, which could ultimately inform future policy development.

## Notes

Supplement 1. eFigure 1. Flow-chart of recruitment and selection of participants eFigure 2. Comparison of methods for measuring meat consumption eFigure 3. Attrition by level of meat consumption eFigure 4: Baseline characteristics of participants by meat consumption eFigure 5: Health status at baseline by total meat consumption and *APOE* genotype eFigure 6: Comparison of time-point(s) of exposure measurement and stratifications on various sub-groups eFigure 7: Validation of our primary dichotomization (*APOE34/44* vs others) eFigure 8: Analysis of potential mediation or confounding by various diet parameters. eFigure 9. Fixed-effects model adjusted for time and cross-sectional baseline analyses eFigure 10: Analysis of cognitive trajectories and dietary changes by dementia status and *APOE* eFigure 11: Cumulative incidence curves for dementia by *APOE* and total meat consumption eFigure 12: Sensitivity analysis excluding individuals with possible cognitive impairment eFigure 13. Noncognitive health outcomes eFigure 14. Sensitivity analyses exploring the role of vitamin B12 status eFigure 15. Analyses by individual food groups eFigure 16. Distribution of consumption from other food groups by quintiles of meat consumption eTable 1. Correlations between diet variables eTable 2. Participant status eTable 3. Baseline characteristics of participants without and with cognitive trajectories eTable 4. Association of meat consumption at baseline with dementia risk over 15 years eMethods 1: Triangulation: within-participant associations and participants with baseline data only eMethods 2: Associations between meat consumption and all-cause mortality eReferences.

Supplement 2.
Data Sharing Statement
