# Supplementary material for: Meat Consumption and Cognitive Health by APOE Genotype
Source: JAMA Netw Open. 2026 Mar 19;9(3):e266489. doi: 10.1001/jamanetworkopen.2026.6489 (PMC13003371; doi:10.1001/jamanetworkopen.2026.6489)
Supplement: Supplement 1. — eFigure 1. Flow-chart of recruitment and selection of participants eFigure 2. Comparison of methods for measuring meat consumption eFigure 3. Attrition by level of meat consumption eFigure 4: Baseline characteristics of participants by meat consumption eFigure 5: Health status at baseline by total meat consumption and APOE genotype eFigure 6: Comparison of time-point(s) of exposure measurement and stratifications on various sub-groups eFigure 7: Validation of our primary dichotomization (APOE34/44 vs others) eFigure 8: Analysis of potential mediation or confounding by various diet parameters. eFigure 9. Fixed-effects model adjusted for time and cross-sectional baseline analyses eFigure 10: Analysis of cognitive trajectories and dietary changes by dementia status and APOE eFigure 11: Cumulative incidence curves for dementia by APOE and total meat consumption eFigure 12: Sensitivity analysis excluding individuals with possible cognitive impairment eFigure 13. Noncognitive health outcomes eFigure 14. Sensitivity analyses exploring the role of vitamin B12 status eFigure 15. Analyses by individual food groups eFigure 16. Distribution of consumption from other food groups by quintiles of meat consumption eTable 1. Correlations between diet variables eTable 2. Participant status eTable 3. Baseline characteristics of participants without and with cognitive trajectories eTable 4. Association of meat consumption at baseline with dementia risk over 15 years eMethods 1: Triangulation: within-participant associations and participants with baseline data only eMethods 2: Associations between meat consumption and all-cause mortality eReferences. [file jamanetwopen-e266489-s001.pdf]

Supplemental Online Content

Norgren J, Carballo-Casla A, Grande G, et al. Meat consumption and cognitive health by *APOE* genotype. *JAMA Netw Open*. 2026;9(3):e266489. doi:10.1001/jamanetworkopen.2026.6489

eFigure 1. Flow-chart of recruitment and selection of participants
eFigure 2. Comparison of methods for measuring meat consumption
eFigure 3. Attrition by level of meat consumption
eFigure 4: Baseline characteristics of participants by meat consumption
eFigure 5: Health status at baseline by total meat consumption and APOE genotype
eFigure 6: Comparison of time-point(s) of exposure measurement and stratifications on various sub-groups
eFigure 7: Validation of our primary dichotomization (APOE34/44 vs others)
eFigure 8: Analysis of potential mediation or confounding by various diet parameters.
eFigure 9. Fixed-effects model adjusted for time and cross-sectional baseline analyses
eFigure 10: Analysis of cognitive trajectories and dietary changes by dementia status and APOE
eFigure 11: Cumulative incidence curves for dementia by APOE and total meat consumption
eFigure 12: Sensitivity analysis excluding individuals with possible cognitive impairment
eFigure 13. Noncognitive health outcomes
eFigure 14. Sensitivity analyses exploring the role of vitamin B12 status
eFigure 15. Analyses by individual food groups
eFigure 16. Distribution of consumption from other food groups by quintiles of meat consumption
eTable 1. Correlations between diet variables
eTable 2. Participant status
eTable 3. Baseline characteristics of participants without and with cognitive trajectories
eTable 4. Association of meat consumption at baseline with dementia risk over 15 years
eMethods 1: Triangulation: within-participant associations and participants with baseline data only
eMethods 2: Associations between meat consumption and all-cause mortality
eReferences.

This supplemental material has been provided by the authors to give readers additional information about their work.

Content:

1. Flow-chart of recruitment and selection of participants (eFigure 1; page 2)
2. Characteristics of participants (eTable 3, eFigure 4-5; page 3–4)
3. Description of diet variables (eTable 1, eFigure 2, eFigure 16; page 5–6)
4. Sensitivity analyses: Association between meat consumption and cognitive outcomes:
    - Various stratifications on subgroups (eFigure 6; page 7)
    - Complete *APOE* stratification across six genotypes (eFigure 7; page 8)
    - Mediation analysis by macronutrient parameters; a comment on BMI (eFigure 8; page 9)
    - Triangulation: within-participant associations and participants with baseline data only (eMethods 1; eFigure 9; page 10)
    - Reverse causation evaluation (eFigure 10; page 11)
5. Time-to-event analyses of dementia risk
    - Cumulative incidence curves for dementia (eTable 2, eFigure 3, 11; page 12)
    - Quintile-based sensitivity analyses (eTable 4; page 13)
    - Linear sensitivity analysis on cognitive intact individuals (eFigure 12; page 14)
6. Post hoc analyses:
    - Associations between meat consumption and all-cause mortality (eMethods 2: eFigure 13; p. 15)
    - Associations between meat consumption and some key biomarkers (eFigure 13; page 15)
    - A potential role of vitamin B12 absorption? (eFigure 14; page 16)
    - Replacement analyses: meat versus individual food groups (eFigure 15; page 17)

7. eReferences (page 18)

The content is thematically organized, while the numbering of figures and tables follows the order of first call-out in the main paper.

© 2026 Norgren J et al. *JAMA Network Open*.

1. Flow-chart of Recruitment and Selection of Participants

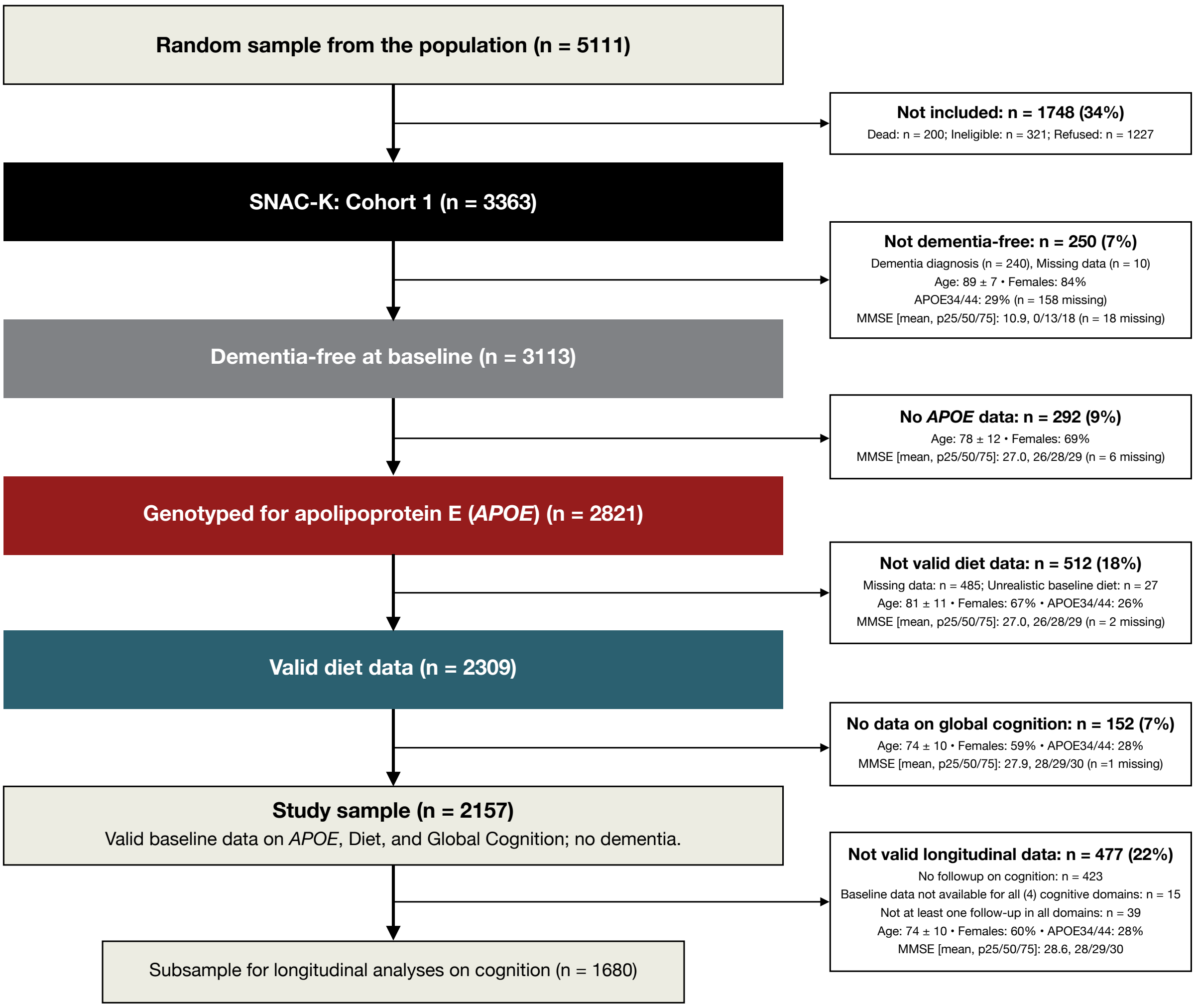

eFigure 1: Overview of the selection of participants.
Percentages excluded refer to the previous stage. MMSE: Mini-Mental Examination score at baseline (max: 30).
Unrealistic baseline diet defined as total energy intake out of range (males: 800–5000 kcal/day; females: 500–4000 kcal/day)

Compared to the study sample (n = 2157), excluded individuals (n = 1206) were older (mean 81.0 versus 71.2), had a lower MMSE score (24.0 versus 29.0; 27 versus 0 missing), more chronic diseases (4.9 versus 3.7), and a larger proportion of females (70% versus 62%). Prevalence of apolipoprotein E genotypes (*APOE*) $\varepsilon3/\varepsilon4$ and $\varepsilon4/\varepsilon4$ (APOE34/44) were similar for the study sample (26.4%) and the excluded group (26.9%); however, 37% of the excluded had missing *APOE* data.

The sub-sample (n = 1680) that was included in the primary (longitudinal) analyses on global cognition did not differ substantially from the full study sample (n = 2157), as shown in **eTable 3**. Within the longitudinal sample (n=1680), 90% had cognitive data at 6-year follow-up and 59% had such data at 12-year follow-up.

© 2026 Norgren J et al. *JAMA Network Open*.

2. Characteristics of Participants

eTable 3. Baseline characteristics of participants without and with cognitive trajectories.

	**Study sample** (n = 2157)	**Primary cognition analyses** (n = 1680)
Age (years)	71.2 (9.2)	70.3 (8.9)
Sex (females/males)	1337/820 (62/38)	1052/628 (63/37)
**Apolipoprotein E genotype**		
APOE $\varepsilon 2/\varepsilon 2$	13 (0.6)	9 (0.5)
APOE $\varepsilon 2/\varepsilon 3$	224 (10)	175 (10)
APOE $\varepsilon 2/\varepsilon 4$	57 (2.6)	48 (2.9)
APOE $\varepsilon 3/\varepsilon 3$	1294 (60)	1013 (60)
APOE $\varepsilon 3/\varepsilon 4$	508 (24)	391 (23)
APOE $\varepsilon 4/\varepsilon 4$	61 (2.8)	44 (2.6)
**Clinical variables**		
Mini-Mental State Examination (MMSE)	29.0 (1.2)	29.1 (1.0)
Global cognition (z-score)	0 (1)	0.13 (0.96)
Chronic diseases (n)	3.7 (2.2)	3.4 (2.1)
Diabetes	166 (7.7)	109 (6.5)
Statins	291 (13.5)	229 (13.6)
Body-mass index (BMI; kg/m^2)	26.0 (4.0)	26.1 (3.9)
Systolic blood pressure (mm Hg)	144 (19)	144 (19)
HbA1c (%)	4.6 (0.7)	4.5 (0.6)
Total cholesterol (mg/dL • mmol/L)	236 (43) • 6.1 (1.1)	236 (43) • 6.1 (1.1)
**Sociodemographic and lifestyle variables**		
Education (years)	12.4 (4.2)	12.7 (4.1)
Living arrangements (not alone)	1066/2150 (50)	803/1675 (50)
Occupation (manual)	396/2150 (18)	283/1675 (17)
Physical activity (low, mid, high)	458, 1141, 558 (21, 53, 26)	315, 895, 470 (19, 53, 28)
Tobacco smoker (current)	279/2144 (13)	192/1670 (11)
**Diet variables**		
Total energy intake (kcal/day)	1970 (651)	1963 (634)
Carbohydrates (digestible, E%)	44 (7)	44 (7)
Fat (E%)	35 (7)	34 (7)
Protein (E%)	14 (2)	14 (2)
Fiber (g)	25 (10)	25 (10)
Alcohol (E%)	4.5 (4.4)	4.7 (4.4)
Total meat (g/14000 kcal)	539 (264)	543 (263)
Red meat, unprocessed (g/14000 kcal)	273 (165)	279 (168)
Poultry, unprocessed (g/14000 kcal)	98 (96)	100 (88)
Processed meat (g/14000 kcal)	168 (134)	164 (132)
Processed-to-total meat ratio (%)	30 (18)	29 (18)
Alternative Healthy Eating Index	62 (10)	62 (9)

Note: Data are expressed as No. (%) for categorical variables and mean values (SD) for continuous variables. The denominator for No. is 2157 or 1680, unless stated differently. Meat intake is expressed as grams per 14,000 kcal (equivalent to weekly intake for a 2000 kcal/day diet; mean intake in the cohort was 1970 kcal/day). Missing data: BMI (n=16), systolic blood pressure (n=4), HbA1c (n=31), total cholesterol (n=33), living arrangements (n=7), occupation (n=7), tobacco smoker (n=13), processed-to-total meat ratio (n=3). E%: percentages of total energy intake

© 2026 Norgren J et al. *JAMA Network Open*.

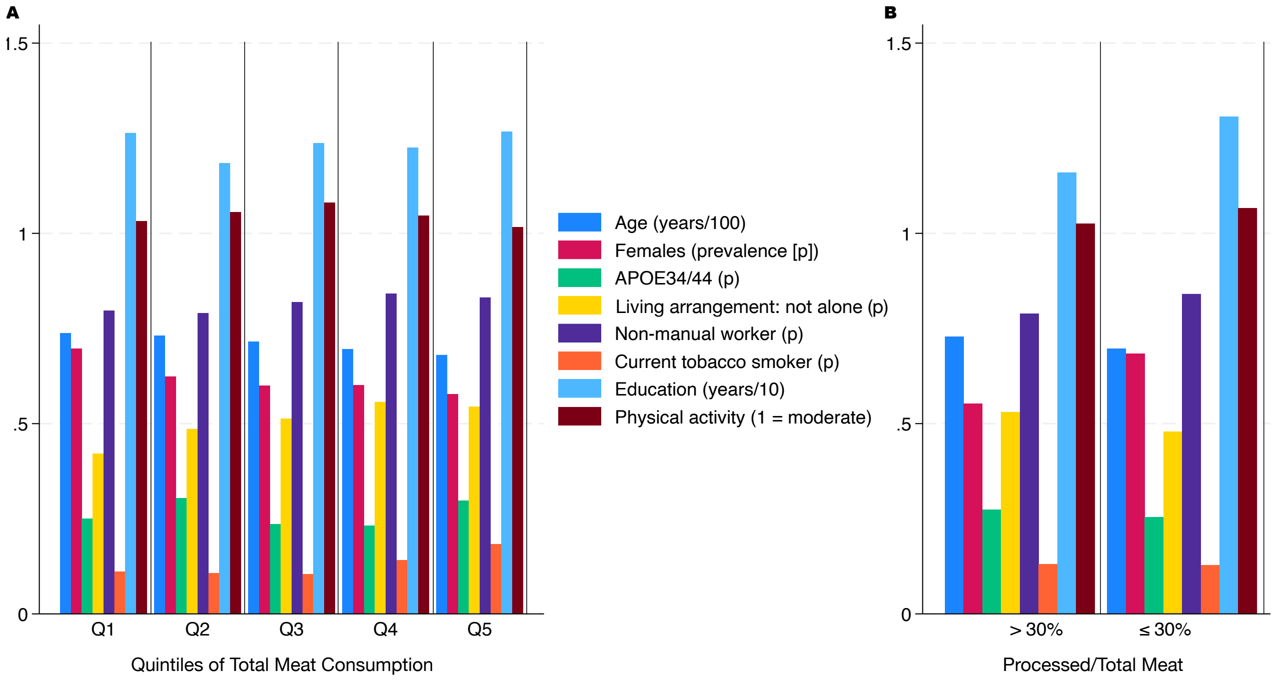

eFigure 4: Baseline characteristics of participants by meat consumption.
A: Total meat (g/total kcal). **B:** The ratio between processed and total meat. The 30% cut-off is very close to the median value. (n = 2157)

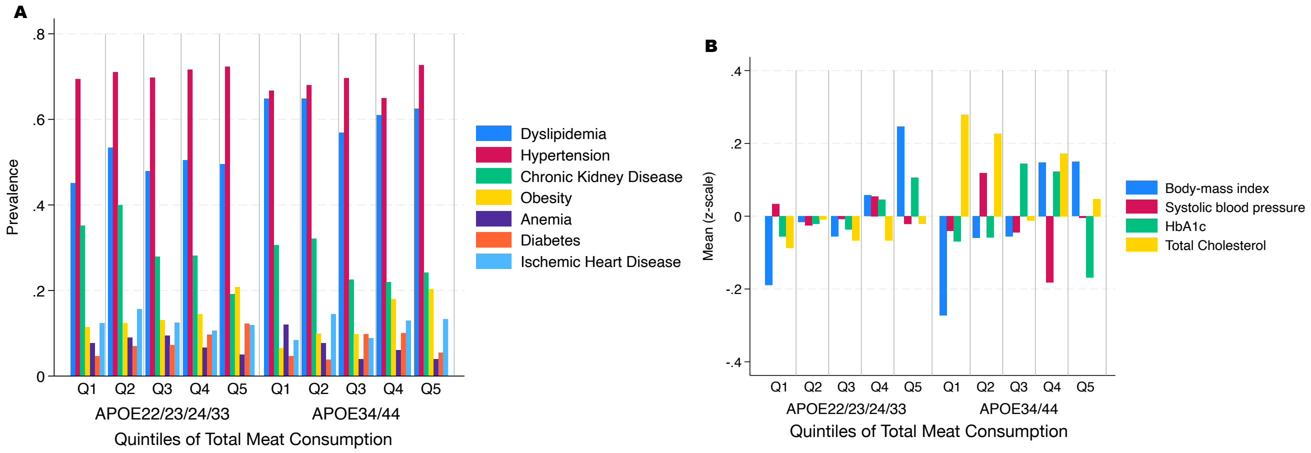

eFigure 5: Health status at baseline by total meat consumption and *APOE* genotype.
A: Prevalence of diagnoses. **B:** Biomarkers: relative comparison of standardized mean values. (n = 2157).

© 2026 Norgren J et al. *JAMA Network Open*.

3. Description of Diet Variables

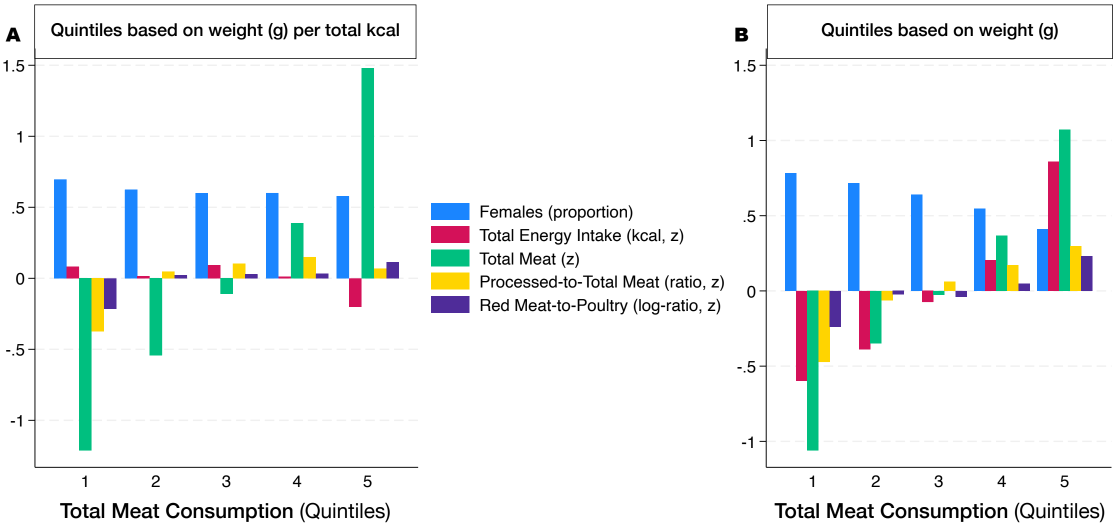

eFigure 2. Comparison of methods for measuring meat consumption.
(A) By weight divided by total energy intake **(B)** By weight.

We apply method A based on the following considerations:

- The biological impact of meat consumption on health is assumed to depend on its *proportion* of diet. For example, 100 g meat represents a very different dietary load in a 1500 kcal/day diet compared with a 3000 kcal/day diet. By dividing by total energy intake (kcal), we obtain a physiologically comparable measure.
- Method B is strongly confounded by sex and total energy intake. Importantly, this issue cannot be solved simply by adjusting for sex and kcal at the between-subject level, because females remain underrepresented in the higher quantiles. Whether to adjust for kcal in the model depends on its role in the causal structure: if total energy intake primarily reflects confounding (selection bias), adjustment may be appropriate; if, however, the compositional aspect of diet (meat g/kcal) influences total energy intake through hunger or satiety, then kcal could be a mediator, in which case adjustment may not be desirable. Retaining flexibility in this decision is important from a causal inference perspective.[5]
- Further arguments for disentangling compositional from quantitative aspects of diet—and for the use of ratios as predictor variables—have been discussed by Corrêa Leite.[6]

In summary, methods A and B address different research questions, and A is more relevant for guiding dietary recommendations. By adjusting for total energy intake at the within-person level, we obtain a more meaningful predictor variable. The sub-distribution of meat types across quintiles is relatively stable in method A, suggesting that the energy density of total meat does not differ substantially. Thus, g/kcal can serve as a proxy for the caloric proportion of meat in the diet.

eTable 1. Correlations between diet variables.

	**Total Meat**	Red Meat	Poultry	Processed Meat	Processed-to-Total Meat Ratio	Red Meat-to-Poultry Ratio	AHEI
Red Meat (unprocessed)	0.82						
Poultry (unprocessed)	0.45	0.20					
Processed Meat	0.63	0.24	–0.07				
**Processed-to-Total Meat Ratio**	**0.14**	–0.20	–0.32	0.74			
**Red Meat-to-Poultry Ratio**	**0.11**	0.31	–0.54	0.22	**0.24**		
AHEI	–0.20	–0.13	0.16	–0.36	–0.36	–0.24	
AHEI, excluding meat items	0.02 [a]	–0.01 [a]	0.15	–0.13	–0.18	–0.17	0.94

Notes: • Correlations between baseline values analyzed by Pearson's *r* (n = 2157)
• Primary and secondary exposures in bold, showing relatively high independence from each other.
• Exploratory exposures may be interpreted with consideration of collinearity with total meat.
AHEI: Alternative Healthy Eating Index; [a] Not statistically significant correlation (P ≥ .39)

© 2026 Norgren J et al. *JAMA Network Open.*

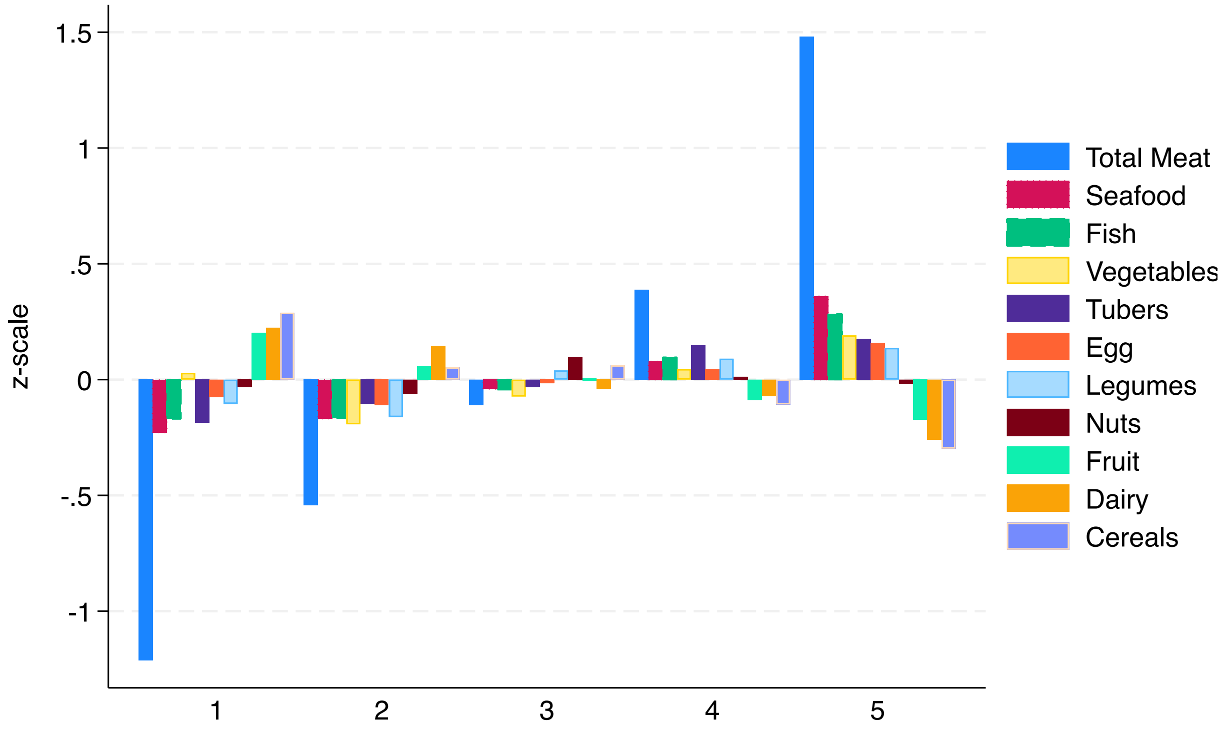

eFigure 16. Distribution of consumption from other food groups by quintiles of total meat consumption.
Baseline values (n = 2157).

© 2026 Norgren J et al. *JAMA Network Open*.

4. Sensitivity analyses: Association between meat consumption and global cognition

These analyses are primarily intended for graphical interpretation.

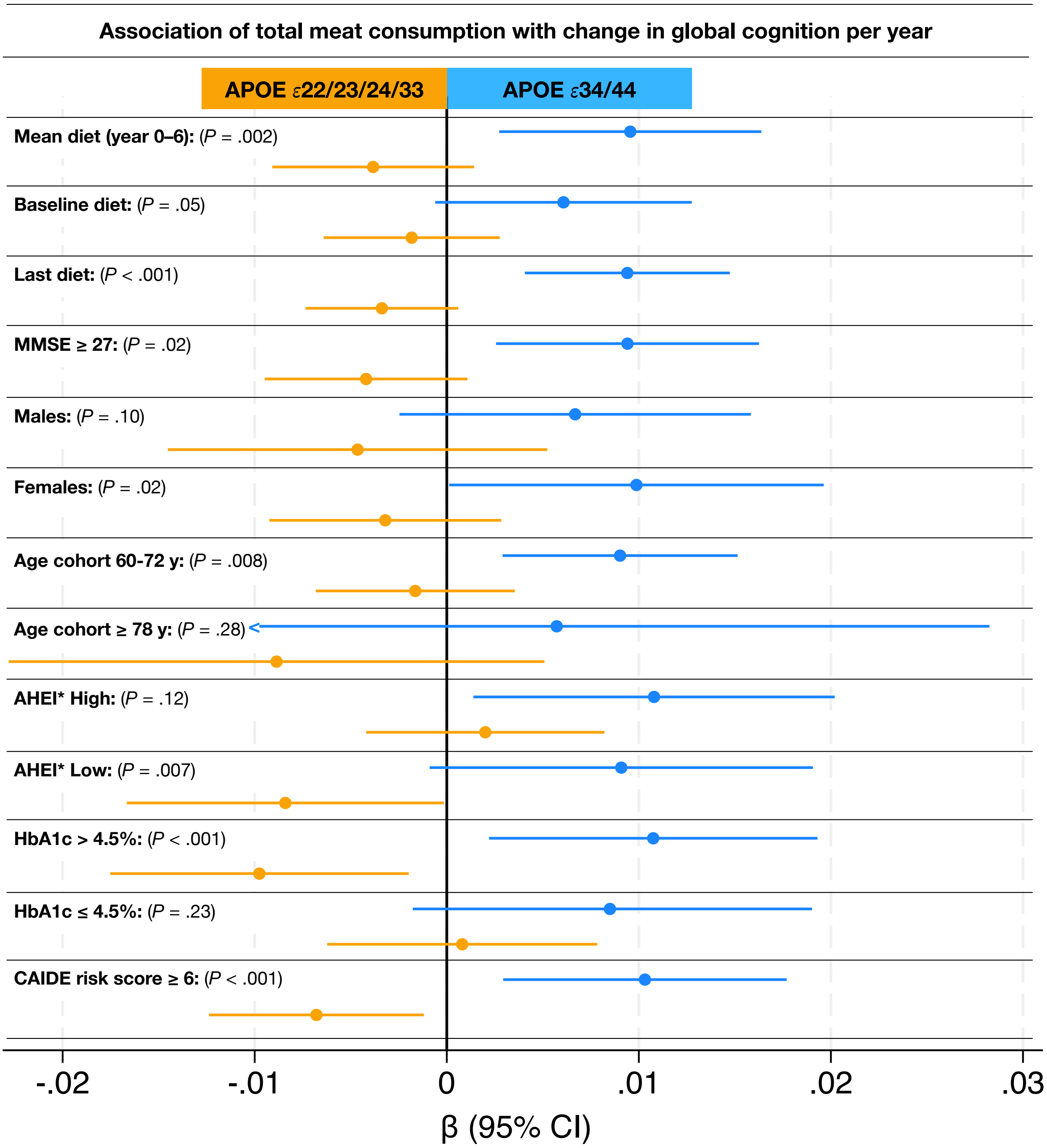

eFigure 6: Comparison of time-point(s) of exposure measurement and stratifications on various sub-groups
The first three rows compare dietary exposure calculated from mean, baseline, and last assessment, respectively.
The following rows use mean diet as the exposure and include sub-samples, as indicated. Linear regression between total meat consumption (g/kcal, z-transformed) and trajectory of global cognition (change in z-score per year), adjusted for age, sex, education, *APOE*, living arrangements, occupation type, physical activity, smoking, alcohol intake, total energy intake, Alternative Healthy Eating Index (*AHEI, calculated without meat items), number of chronic diseases, and baseline cognition. *P*-values for interaction *meat* x *APOE* are indicated. MMSE: Mini-Mental State Examination. CAIDE risk score (Cardiovascular Risk Factors, Aging and Dementia) calculated according to Kivipelto & Ngandu (2016)[7]; the ≥6 cut-off aligns with inclusion criteria of the FINGER trial.[8] Cut-offs for AHEI and HbA1C are median-split.

A post hoc analysis with males and females in the same model found no interaction by diet × sex in any *APOE* strata ($P \geq .67$) or a 3-way interaction diet × sex × *APOE* ($P = .99$).

© 2026 Norgren J et al. *JAMA Network Open*.

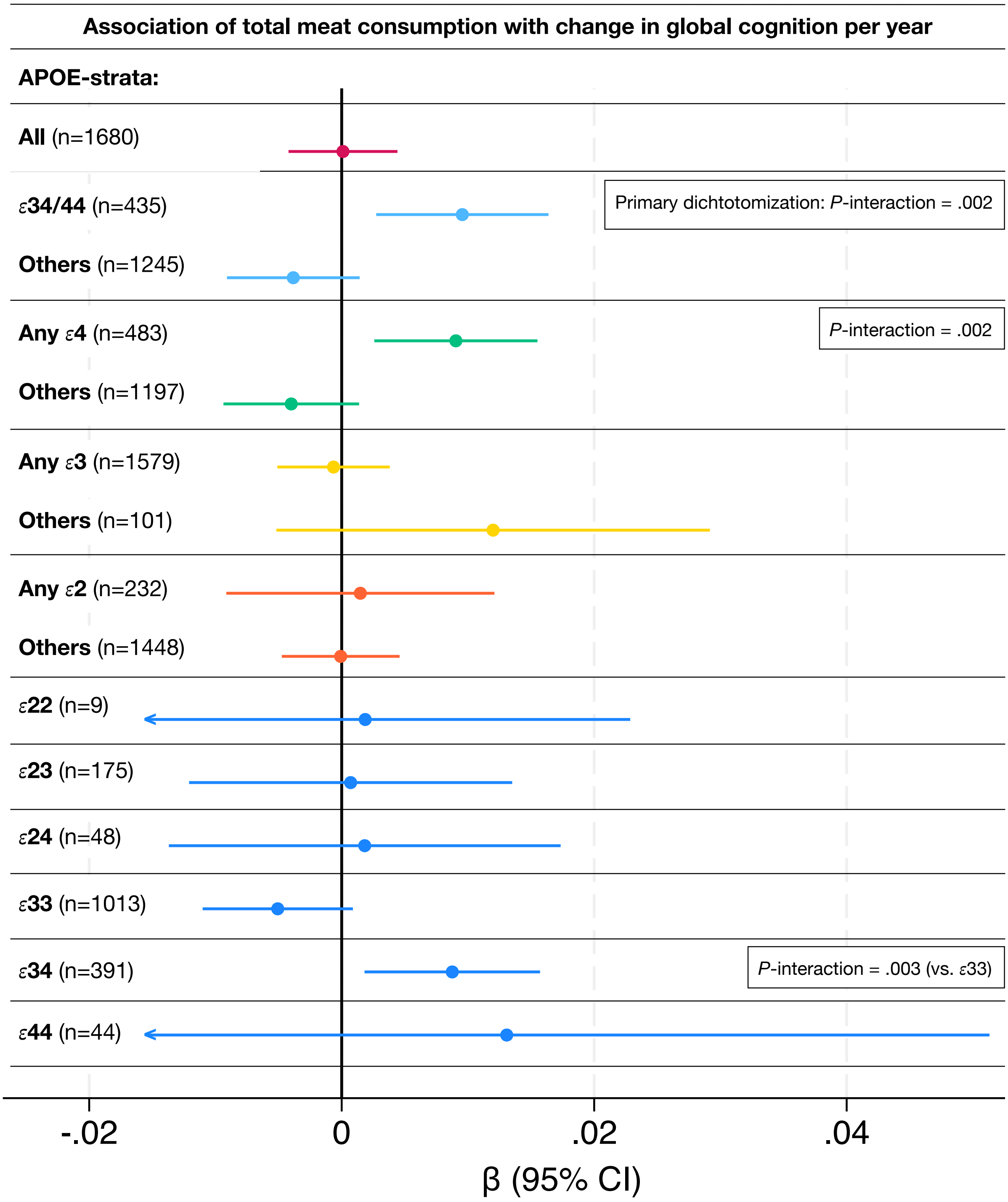

eFigure 7: Validation of our primary dichotomization (APOE34/44 vs others).
Linear regression between total meat consumption (1 SD increment) and trajectory of global cognition (change in z-score per year), adjusted for age, sex, education, *APOE*, living arrangements, occupation type, physical activity, current smoking status, alcohol intake, total energy intake, Alternative Healthy Eating Index (calculated without meat items), number of chronic diseases, and baseline cognition. Key *P*-values for the interaction *meat exposure* × *APOE* are indicated.

© 2026 Norgren J et al. *JAMA Network Open*.

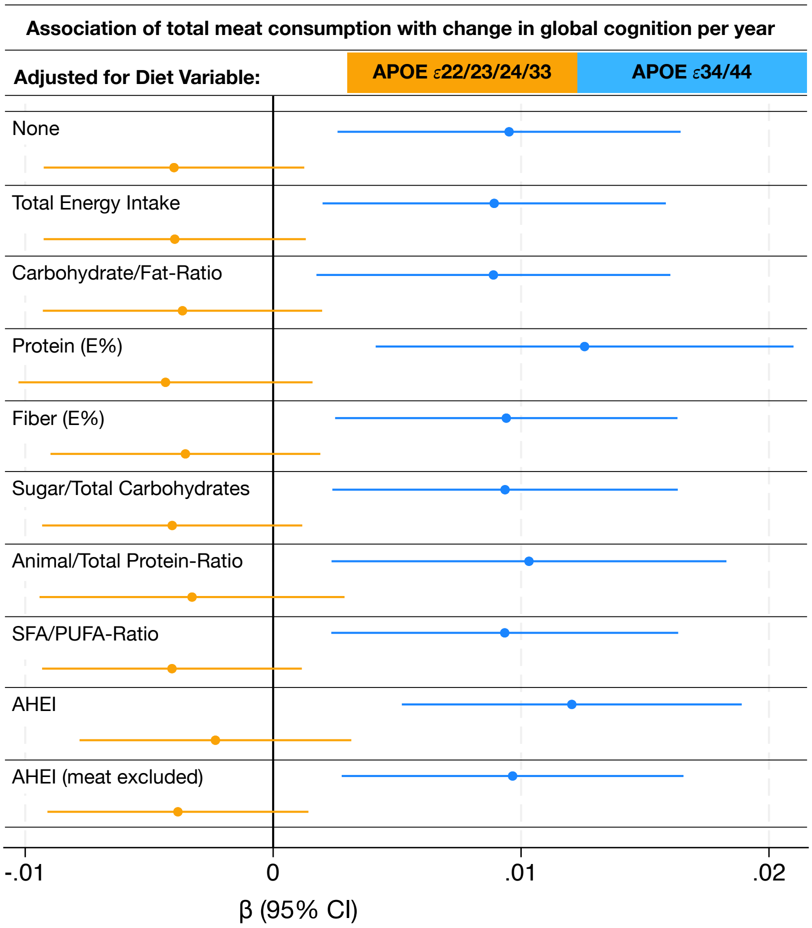

eFigure 8: Analysis of potential mediation or confounding by various diet parameters.
Linear regression between total meat consumption (z-scale) and trajectory of global cognition (change in z-score per year), adjusted for age, sex, education, *APOE*, physical activity, and baseline cognition.
SFA/PUFA: saturated/polyunsaturated fatty acids, AHEI: Alternative Healthy Eating Index

Interpretation of eFigure 8: Total energy intake (TEI) and AHEI (meat items excluded) were included as potential confounders in the primary model, but their inclusion had negligible impact. We acknowledge that TEI could alternatively act as a mediator, if the dietary proportion of meat vs other food groups influences satiety or hunger; however, its impact on the estimates was minimal. Likewise, the macronutrient parameters explored as potential mediators did not appear to play this role. The slight shift in point estimates after protein adjustment was not in the direction that would suggest mediation.

A comment on body-mass index (BMI):

We did not primarily consider BMI as a confounder but rather a possible mediator. However, the association between BMI and cognitive outcomes may be modified by age, which may complicate interpretations. After concluding that exploratory adjustment for BMI had negligible impact on the results, it was not further considered for inclusion in the models.

© 2026 Norgren J et al. *JAMA Network Open*.

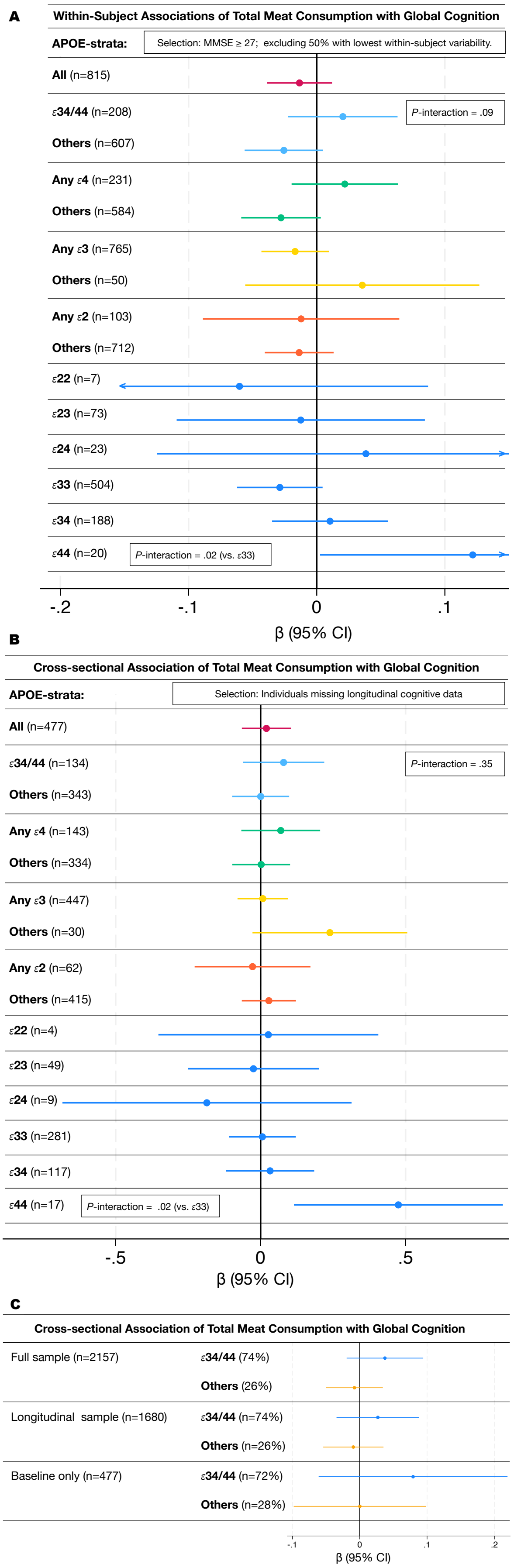

eMethods 1: Triangulation

The rationale and application of disentangling between- and within-subject associations have been described previously.[1,2] We assume that intra-individual variability in self-reported diet reflects a combination of (A) random fluctuations around a long-term habit, and (B) true changes sufficient to impact health outcomes. The target trial[3] for the between-subject analyses would be a parallel-group trial starting at baseline, reflecting perspective A. Thus, the mean dietary value over time serves as the long-term assignment (assumed to reduce measurement error). The within-subject analysis reflects perspective B and is conceptualized as a crossover trial. Here, time-updated diet is regressed on time-updated cognition (order-independent, but adjusted for time), excluding subjects with minimal within-variability in diet (defined by median split) and possible cognitive impairment (Mini-Mental State Examination [MMSE] < 27 or dementia within 3 years). The third angle of triangulation targets subjects without longitudinal data.

Results & Interpretation:

In two non-overlapping selections of the sample (**eFigure 9A-B**), APOE44 consistently had the most positive slope—in line with the primary analyses in eFigure 7—between total meat and global cognition, with an interaction effect relative to APOE33. The likelihood that APOE44 by chance would have the largest beta-coefficient three times is $1/6^3 = 0.5\%$.

For our primary stratification—APOE34/44 vs. others— in **eFigure 9**, the direction of estimates and *APOE* interaction aligned with the main results, although the estimates were not statistically significant.

eFigure 9. (A) Fixed-effects model adjusted for time (stratified by *APOE*). Confounding by time-invariant factors may be excluded.[4] **(B-C) Cross-sectional baseline analyses** by exploratory and primary APOE stratifications, respectively. Linear regression adjusted for age, sex, *APOE*, education, living arrangements, occupation type, physical activity, current smoking status, alcohol intake, total energy intake, Alternative Healthy Eating Index (calculated without meat items), and number of chronic diseases. *P*-values for the meat x *APOE* interaction shown for some key comparisons.

© 2026 Norgren J et al. *JAMA Network Open.*

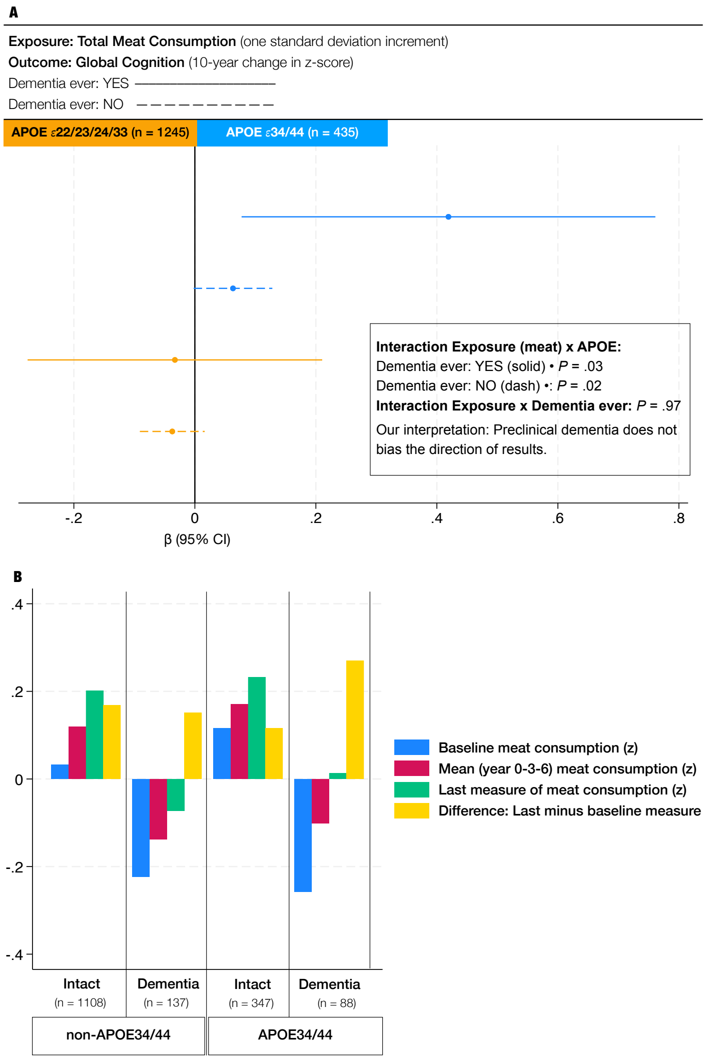

eFigure 10: Analysis of cognitive trajectories and dietary changes by dementia status and APOE

- **Panel A:** Regardless of whether the participants developed dementia within 15 years or not, higher meat consumption was favorably associated with the cognitive trajectory among APOE34/44, and there was a significant interaction effect versus non-APOE34/44.
- **Panel B:** Those who developed dementia had lower meat consumption at baseline. If this was due to a declining trend caused by preclinical disease, we would expect the declining trend to continue within the study. However, meat consumption increased from the first to the last follow-up (yellow bars) regardless of strata.
- **Conclusion:** Both panel A and B suggest that reverse causation is unlikely.

© 2026 Norgren J et al. *JAMA Network Open*.

5. Time-to-Event Analyses on Dementia Risk

These analyses are complementary to Figure 4A and eTable 4. Visual inspection of **eFigure 11** did not suggest major violations of the proportionality assumption. **eFigure 3** indicated non-systematic attrition in relation to the level of meat consumption. Dementia onset was defined as the midpoint between the last known dementia-free day and the day of diagnosis Time zero was set at baseline and exit was defined as the earliest occurrence of dementia incidence, non-dementia death, or—for dementia-free survivors—day after the last follow-up. Exit was truncated to 15.5 years from baseline for n=37—all dementia-free—who had their last follow-up delayed beyond that time-point, to mitigate potential selection bias.

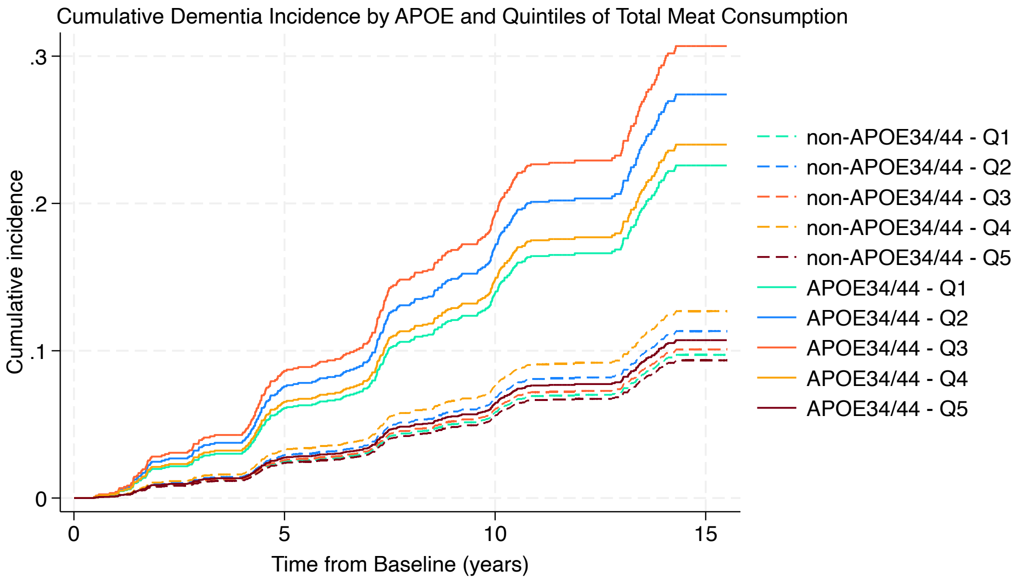

eFigure 11: Cumulative incidence curves for dementia by APOE and total meat consumption. Fine & Gray method, with non-dementia death treated as a competing risk, adjusted for age, sex, education, *APOE*, living arrangements, occupation type, physical activity, current smoking status, alcohol intake, total energy intake, Alternative Healthy Eating Index (*AHEI, recalculated without meat items), and number of chronic diseases.

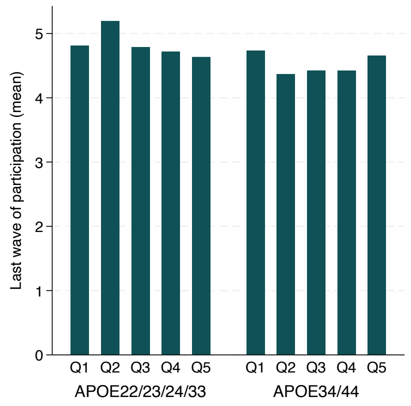

eFigure 3. Attrition by level of meat consumption. Mean value of the last wave of participation shown by quintiles (Q) of total meat consumption and *APOE*. The study included 6 waves of assessment.

		Quintiles of total meat consumption					
**Status at 15.5 years from baseline**	**APOE**	**Q1**	**Q2**	**Q3**	**Q4**	**Q5**	**Total**
**Dementia incidence**	**34/44**	24	35	29	19	10	117
	**non-34/44**	41	43	34	37	24	179
	**Total**	65	78	63	56	34	296
**Non-dementia death**	**34/44**	32	39	26	22	28	147
	**non-34/44**	129	119	115	85	95	543
	**Total**	161	158	141	107	123	690
**All-cause death**	**34/44**	53	64	49	39	35	240
	**non-34/44**	160	153	140	112	119	684
	**Total**	213	217	189	151	154	924
**Dementia-free survival**	**34/44**	52	57	47	59	90	305
	**non-34/44**	154	138	181	209	184	866
	**Total**	206	195	228	268	274	1171
**Total** (excluding all-cause death)	**34/44**	108	131	102	100	128	569
	**non-34/44**	324	300	330	331	303	1588
	**Total**	432	431	432	431	431	2157

eTable 2. Participant status

© 2026 Norgren J et al. *JAMA Network Open*.

eTable 4. Association of Meat Consumption at Baseline with Dementia Risk over 15 Years

		Subdistribution Hazard Ratios for Quintiles of Meat Consumption				
		**Q1**	**Q2**	**Q3**	**Q4**	**Q5**
Total Meat	APOE	(247 g/week)	(397 g/week)	(511 g/week)	(639 g/week)	(869 g/week)
All (n = 2157)	44/34	2.2 (*P* = .04)	2.8 (*P* = .005)	3.2 (*P* = .002)	2.4 (*P* = .03)	1 (ref.)
	others	1.0 (*P* = .86)	1.2 (*P* = .44)	1.1 (*P* = .77)	1.4 (*P* = .21)	1 (ref.)
Excluding dementia ≤ 3 years and MMSE < 27 (n = 2044)	44/34	3.2 (*P* = .006)	2.9 (*P* = .009)	3.8 (*P* = .001)	2.4 (*P* = .05)	1 (ref.)
	others	1.3 (*P* = .42)	1.4 (*P* = .33)	1.3 (*P* = .45)	1.7 (*P* = .10)	1 (ref.)
Males (n = 820)	44/34	1.1 (*P* = .85)	2.4 (*P* = .11)	2.7 (*P* = .09)	1.7 (*P* = .43)	1 (ref.)
	others	0.7 (*P* = .47)	1.0 (*P* = .96)	1.2 (*P* = .65)	1.9 (*P* = .10)	1 (ref.)
Females (n = 1337)	44/34	3.1 (*P* = .03)	3.1 (*P* = .02)	3.6 (*P* = .01)	3.3 (*P* = .03)	1 (ref.)
	others	1.2 (*P* = .53)	1.4 (*P* = .35)	1.1 (*P* = .75)	1.2 (*P* = .55)	1 (ref.)
Unprocessed Red Meat	APOE	(94 g/week)	(188 g/week)	(252 g/week)	(320 g/week)	(474 g/week)
All (n = 2157)	44/34	1.8 (*P* = .10)	1.6 (*P* = .16)	1.6 (*P* = .17)	1.7 (*P* = .12)	1 (ref.)
	others	1.2 (*P* = .49)	1.0 (*P* = .93)	1.0 (*P* = .94)	0.9 (*P* = .68)	1 (ref.)
Poultry	APOE	(3 g/week)	(59 g/week)	(80 g/week)	(108 g/week)	(176 g/week)
All (n = 2157)	44/34	2.0 (*P* = .04)	1.3 (*P* = .45)	1.3 (*P* = .45)	1.6 (*P* = .18)	1 (ref.)
	others	0.9 (*P* = .83)	1.2 (*P* = .41)	0.9 (*P* = .76)	0.8 (*P* = .33)	1 (ref.)
Processed Meat	APOE	(16 g/week)	(92 g/week)	(145 g/week)	(206 g/week)	(332 g/week)
All (n = 2157)	44/34	1.0 (*P* = 92)	1.3 (*P* = .38)	1.1 (*P* = .78)	1.3 (*P* = .34)	1 (ref.)
	others	0.8 (*P* = .30)	1.0 (*P* = .90)	1.2 (*P* = .48)	1.2 (*P* = .57)	1 (ref.)

Note: Quintile assignment based on weight per total energy intake. Median values by g/week are standardized for 2000 kcal/day of total intake. Fine & Gray method was used, with non-dementia mortality as a competing risk. Sensitivity analyses were conducted excluding individuals with dementia diagnosis within 3 years, and Mini-Mental State Examination (MMSE) scores < 27. Adjusted for age, sex, APOE, living arrangements, occupation type, physical activity, current smoking status, alcohol intake, total energy intake, Alternative Healthy Eating Index (AHEI, calculated without meat items), and number of chronic diseases.

The primary comparison was between extreme quintiles, with Q5 chosen as the reference here (in contrast to Q1 in the results text) to illustrate a suggested threshold between Q4 and Q5. Ancillary analyses indicated that the APOE34/44 subdistribution hazard ratio for Q3 (3.2) did not differ from Q1 (*P* = .22), Q2 (*P* = .62), or Q4 (*P* = .75). The implied sex interaction among APOE34/44 for Q5 vs Q1 was not statistically significant (*P* = .15).

© 2026 Norgren J et al. *JAMA Network Open*.

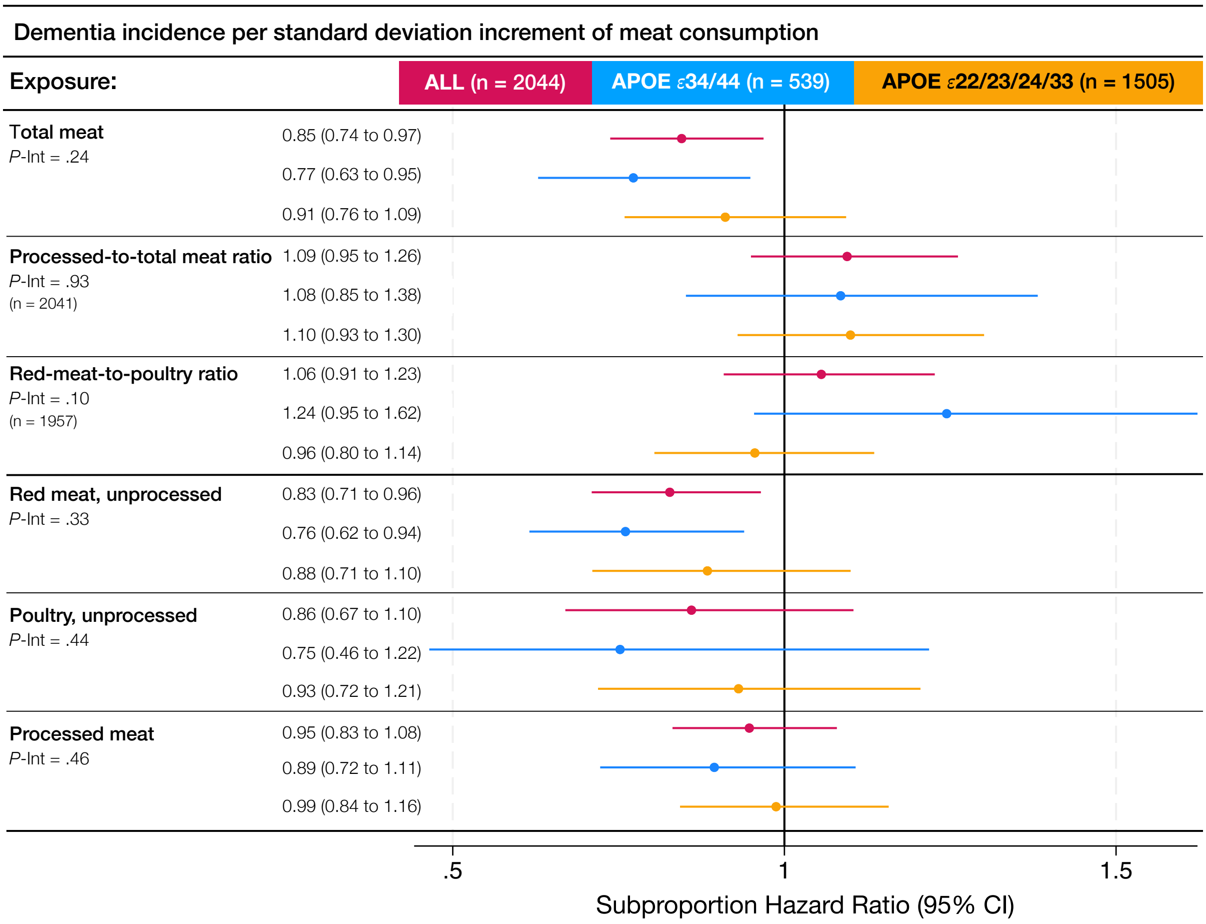

eFigure 12: Sensitivity analysis excluding individuals with possible cognitive impairment.
The analyses correspond to Figure 4A but exclude individuals with possible cognitive impairment (baseline Mini-Mental State Examination (MMSE) < 27 or dementia onset within 3 years from baseline). Estimates by different meat variables as the exposure. Linear regression between meat exposure and cognitive trajectories, adjusted for age, sex, education, *APOE*, living arrangements, occupation type, physical activity, current smoking status, alcohol intake, total energy intake, Alternative Healthy Eating Index (calculated without meat items), and number of chronic diseases.
P-values for interaction between meat variables and *APOE* are shown. One standard deviation equals the following consumption levels—standardized for 2000 kcal/day intake: Total meat: 264 g/week, Unprocessed red meat: 165 g/week, Poultry: 96 g/week, Processed meat: 134 g/week.

© 2026 Norgren J et al. *JAMA Network Open*.

6. Post hoc analyses

eMethods 2: Associations between meat consumption and all-cause mortality

To examine competing risks and whether the *APOE* interactions observed in our cognitive findings extended to other health outcomes, associations between baseline meat consumption and mortality was analyzed by Cox regression. The proportional hazards assumption was assessed using Schoenfeld residuals. Because violations were seen for age, sex, and physical activity, Cox models were stratified by these variables. Proportional hazards assumptions were subsequently evaluated for the remaining covariates and were not violated. Complementary to the hazard ratios (HR) reported in Figure 4B, Kaplan-Meier survival curves are shown in **eFigure 13A** and **B**, excluding deaths within 1 and 6 years, respectively. Estimates remained similar when the exclusion interval changed from 1 to 6 years; e.g., for unprocessed meat, estimates for APOE34/44 changed from HR = 0.85; *P* = 0.04; *P*-Interaction = .03 to HR = 0.86; *P* = 0.08; *P*-Interaction = .04. Some key health biomarkers were additionally studied, including total cholesterol, which was the only available lipid marker (**eFigure 13C**).

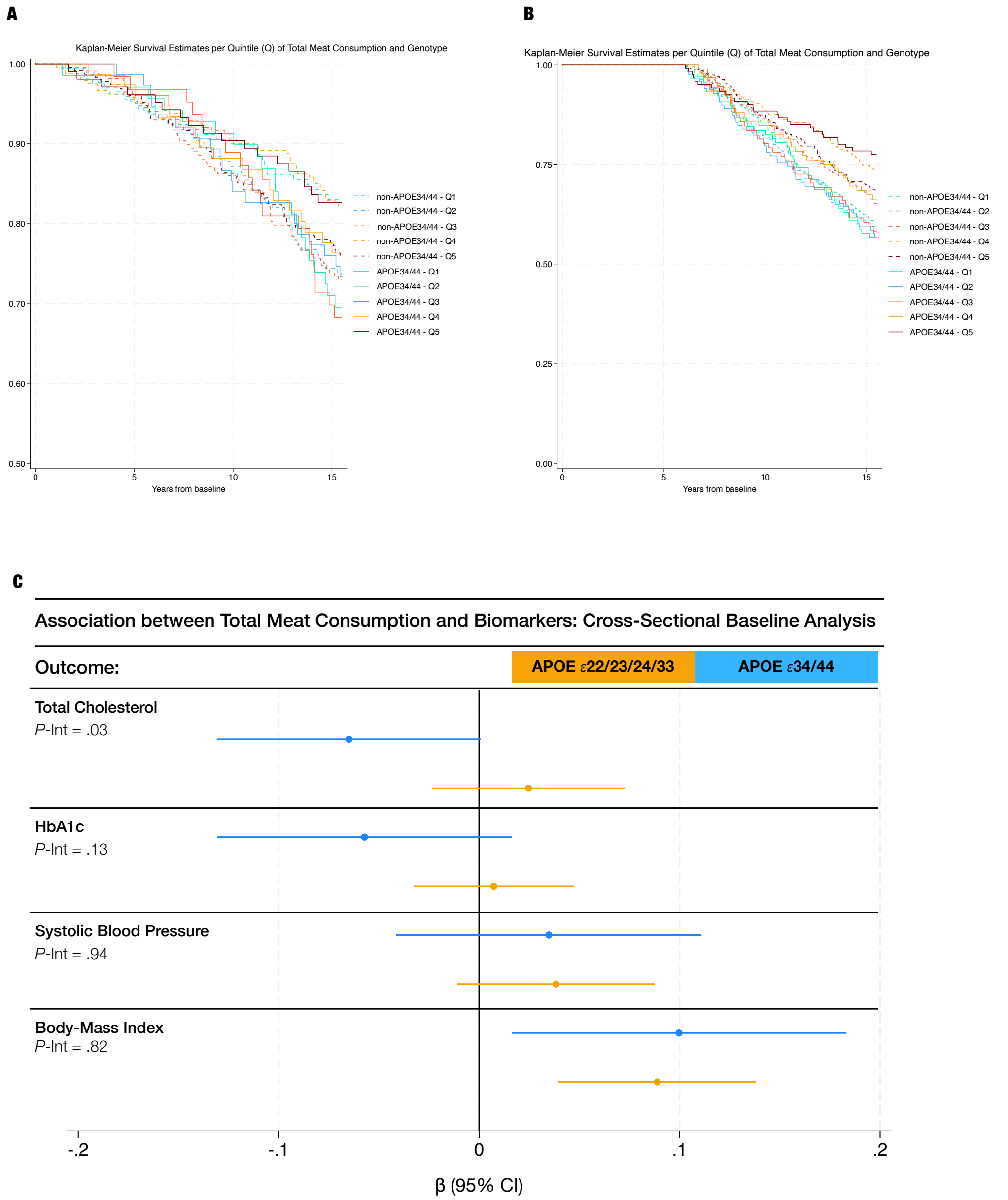

eFigure 13. Non-cognitive health outcomes.

(A) Kaplan-Meier survival estimates for all-cause mortality, excluding deaths within one year (n = 2141).

(B) Similar to (A) but excluding deaths within 6 years (n = 1895).

(C) Biomarker outcomes. Linear regression adjusted for age, sex, physical activity, current smoking status, statin use, diabetes and total energy intake. *P*-Int: *P*-values for interaction by *APOE*

© 2026 Norgren J et al. *JAMA Network Open.*

We investigated how meat quality—defined by the processed-to-total meat ratio with median split—and vitamin B12 status may modify our findings, as displayed in **eFigure 14A-C**. Taken together with an analysis suggesting *APOE* dependent vitamin B12 absorption (**eFigure 14D**), we speculate that the food matrix[9] may play a role: Among APOE34/44, vitamin B12 appear to be absorbed more effectively from meat than from other sources. As a sensitivity analysis indicated an interaction for APOE33 versus APOE2 carriers, we report three APOE strata.

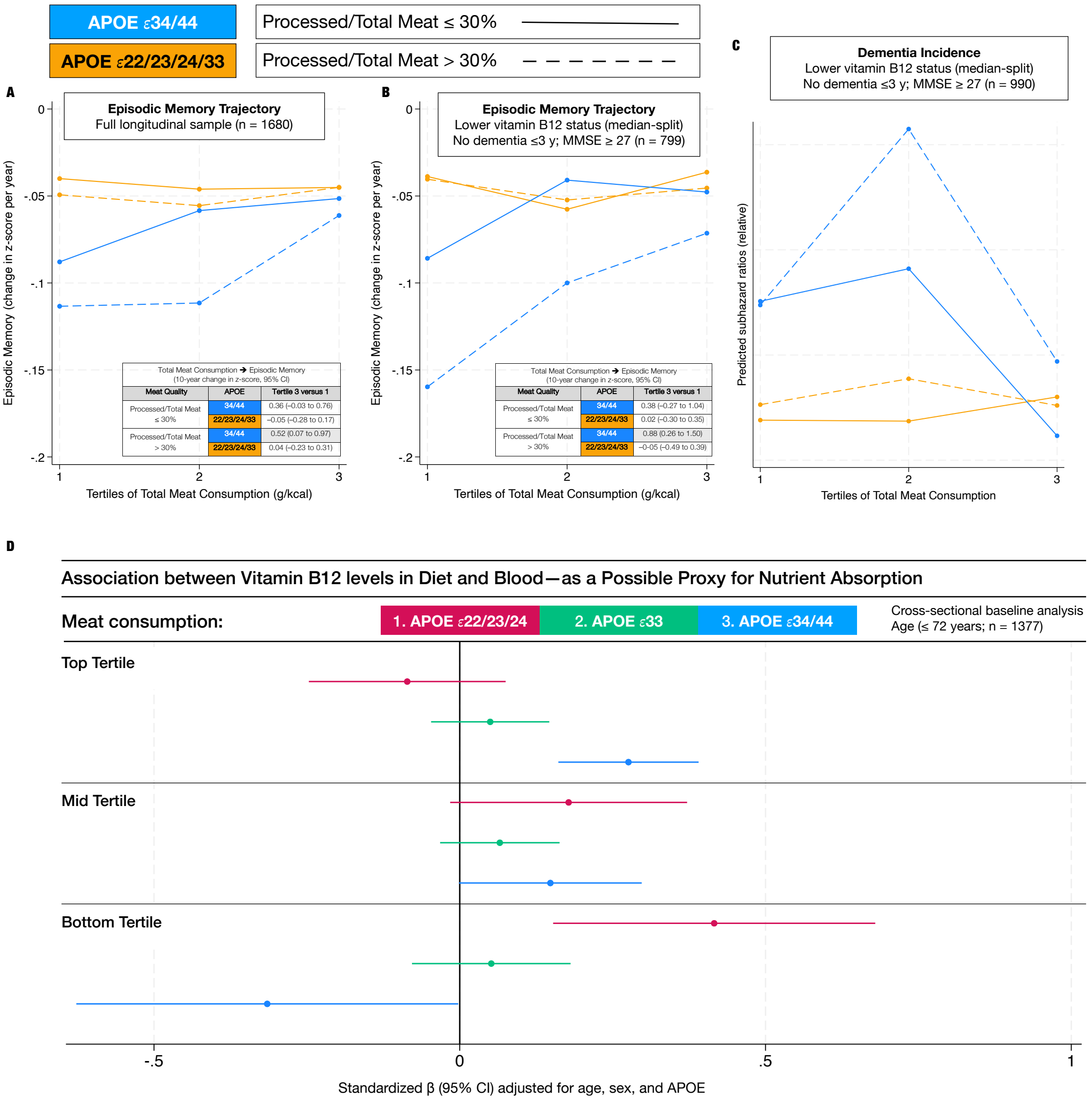

Our interpretation of panel D:

Linear effect modification of the association between dietary and circulating vitamin B12 (a proxy for nutrient absorption) by 1 SD higher meat intake:

1. APOE2-carriers: β = –0.15 (P < 0.001) • Worse absorption from meat compared to other sources.
2. APOE33: β = –0.01 (P = 0.80) • "Metabolic flexibility" = positive slope between vitamin B12 in diet and blood — regardless of meat level.
3. APOE34/44: β = +0.22 (P = 0.001) • Better absorption from meat compared to other sources.

3-way interaction for *APOE* x Meat consumption (linear) x Dietary Vitamin B12 regressed on Circulating Vitamin B12:
• APOE33 vs. APOE34/44: P = 0.002 • APOE33 vs. APOE2-carriers: P < 0.001

eFigure 14. Sensitivity analyses exploring the role of vitamin B12 status. **(A-C)** Cognitive outcomes analyzed in adjusted models, similar to the main analyses, targeting the impact of meat quality. In (B) and (C), subjects with possible cognitive impairment—defined by dementia onset within 3 years or MMS < 27—were excluded
(D) Associations between dietary and circulating vitamin B12 levels, examined as a possible proxy for nutrient absorption (although this cannot be distinguished from utilization and clearance). Age cohorts >72 excluded to mitigate survival bias.

© 2026 Norgren J et al. *JAMA Network Open*.

In addition to our primary research question—contrasting meat consumption with all other food groups combined—we conducted secondary analyses in which meat was contrasted with individual food groups. Log-ratios between meat consumption and the respective replacement food group were used as exposure variables, consistent with approaches proposed for compositional data analysis.[6]

Food group levels were truncated at the 1st percentile of their food-specific baseline distributions, with zero and near-zero values set to this threshold (all values >0). Values above the 99th percentile were similarly truncated to reduce the influence of extreme intakes. Log-ratios were subsequently standardized using baseline distributions, and means across follow-up assessments were used as exposure variables. Associations with cognitive trajectories were then examined (**Figure e15).**

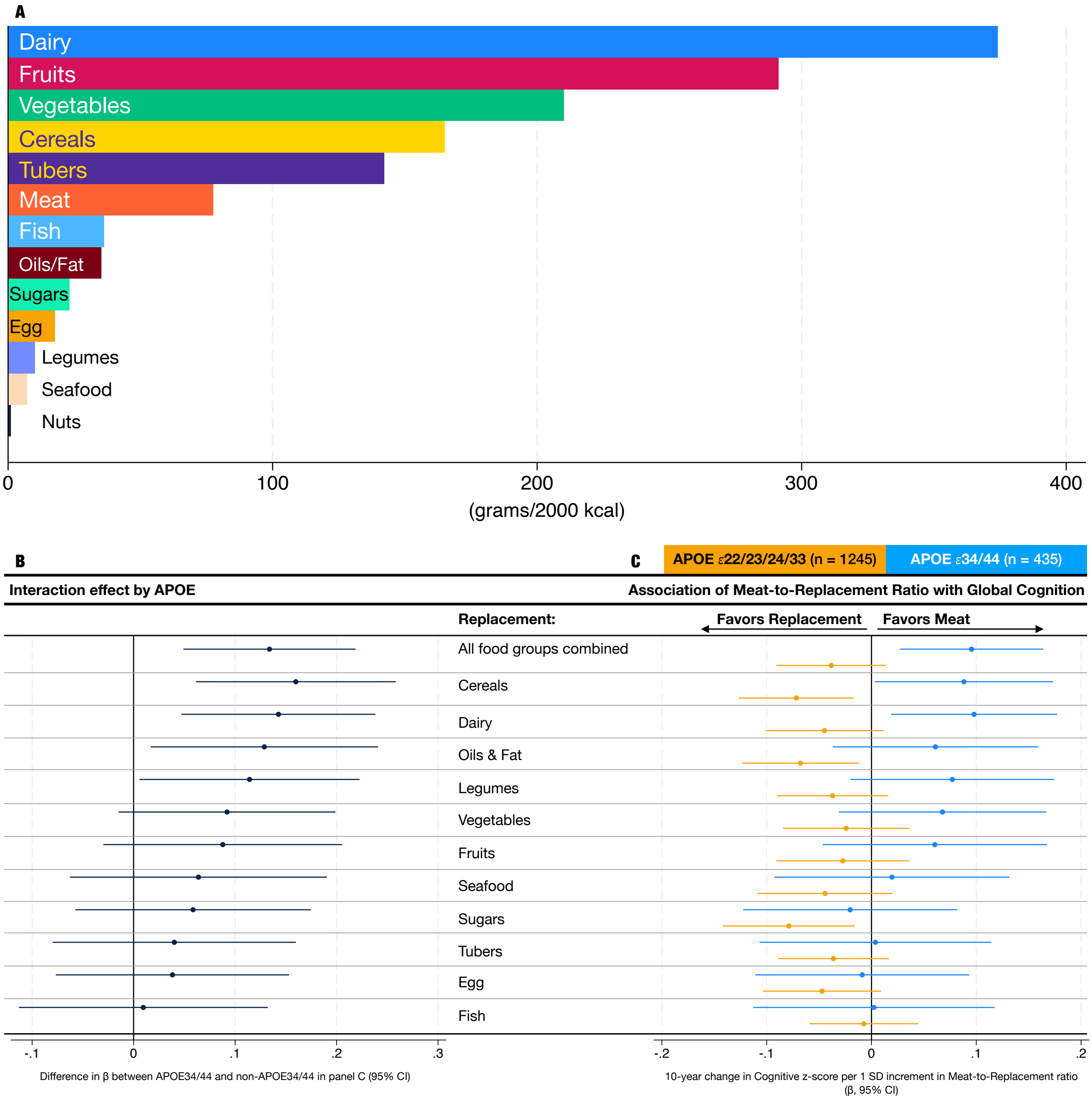

eFigure 15. Analyses by individual food groups. (A) Baseline distributions of mean consumption levels. **(B)** Interaction effects by APOE genotype, corresponding to the food replacement analyses shown in panel **(C)**. Results from primary analysis (top), followed by log-ratio analyses contrasting meat with individual food groups, ordered by decreasing magnitude of the *APOE* interaction. Linear regression adjusted for age, sex, education, *APOE*, living arrangements, occupation type, physical activity, current smoking status, alcohol intake, total energy intake, Alternative Healthy Eating Index (AHEI, recalculated without meat items), number of chronic diseases, and baseline cognition. Tubers primarily refer to potatoes. Nuts were excluded from linear analyses due to very low consumption levels and a highly skewed distribution.

© 2026 Norgren J et al. *JAMA Network Open*.

7. eReferences

1. Norgren J, Sindi S, Sandebring-Matton A, Ngandu T, Kivipelto M, Kåreholt I. The Dietary Carbohydrate/Fat-Ratio and Cognitive Performance: Panel Analyses in Older Adults at Risk for Dementia. *Curr Dev Nutr*. Jun 2023;7(6):100096. doi:10.1016/j.cdnut.2023.100096
2. van de Pol MV, Wright J. A simple method for distinguishing within- versus between-subject effects using mixed models. *Animal Behaviour*. Mar 2009;77(3):753-758. doi:10.1016/j.anbehav.2008.11.006
3. Matthews AA, Danaei G, Islam N, Kurth T. Target trial emulation: applying principles of randomised trials to observational studies. *BMJ (Clinical research ed)*. Aug 30 2022;378:e071108. doi:10.1136/bmj-2022-071108
4. Gunasekara FI, Richardson K, Carter K, Blakely T. Fixed effects analysis of repeated measures data. *International journal of epidemiology*. Feb 2014;43(1):264-9. doi:10.1093/ije/dyt221
5. Franks DW, Ruxton GD, Sherratt T. Ecology needs a causal overhaul. *Biol Rev Camb Philos Soc*. May 9 2025;doi:10.1111/brv.70029
6. Corrêa Leite ML. Log-ratio transformations for dietary compositions: numerical and conceptual questions. *J Nutr Sci*. 2021;10:e97. doi:10.1017/jns.2021.93
7. Kivipelto M, Ngandu T. From Heart Health to Brain Health: Legacy of the North Karelia Project for Dementia Research. *Global heart*. Jun 2016;11(2):235-42. doi:10.1016/j.gheart.2016.04.013
8. Ngandu T, Lehtisalo J, Solomon A, et al. A 2 year multidomain intervention of diet, exercise, cognitive training, and vascular risk monitoring versus control to prevent cognitive decline in at-risk elderly people (FINGER): a randomised controlled trial. *Lancet (London, England)*. Jun 06 2015;385(9984):2255-63. doi:10.1016/s0140-6736(15)60461-5
9. Aguilera JM. The food matrix: implications in processing, nutrition and health. *Crit Rev Food Sci Nutr*. 2019;59(22):3612-3629. doi:10.1080/10408398.2018.1502743

© 2026 Norgren J et al. *JAMA Network Open*.
