# Supplementary material for: Meat Consumption and Cognitive Health by APOE Genotype
Source: JAMA Netw Open. 2026 Mar 19;9(3):e266489. doi: 10.1001/jamanetworkopen.2026.6489 (PMC13003371; doi:10.1001/jamanetworkopen.2026.6489)
Supplement: Supplement 2. — Data Sharing Statement [file jamanetwopen-e266489-s002.pdf]

Data Sharing Statement

Norgren. Meat Consumption and Cognitive Health by *APOE* Genotype. *JAMA Netw Open*. Published online March 19, 2026. doi:10.1001/jamanetworkopen.2026.6489

Data

Data available: Yes
Data types: Other (please specify)
Additional Information: SNAC-K data are available on reasonable request to researchers with approval from the SNAC-K data management and maintenance committee.
How to access data: For further information on this database and on data availability, please refer to https://www.snac-k.se/for-researchers/.
When available: With publication

Supporting Documents

Document types: Other (please specify)
Additional Information: SNAC-K data are available on reasonable request to researchers with approval from the SNAC-K data management and maintenance committee.
How to access documents: SNAC-K data are available on reasonable request to researchers with approval from the SNAC-K data management and maintenance committee. For further information on this database and on data availability, please refer to https://www.snac-k.se/for-researchers/.
When available: With publication

Additional Information

Who can access the data: SNAC-K data are available on reasonable request to researchers with approval from the SNAC-K data management and maintenance committee. For further information on this database and on data availability, please refer to https://www.snac-k.se/for-researchers/.
Types of analyses: SNAC-K data are available on reasonable request to researchers with approval from the SNAC-K data management and maintenance committee. For further information on this database and on data availability, please refer to https://www.snac-k.se/for-researchers/.
Mechanisms of data availability: SNAC-K data are available on reasonable request to researchers with approval from the SNAC-K data management and maintenance committee. For further information on this database and on data availability, please refer to https://www.snac-k.se/for-researchers/.
